# Angelman Syndrome Protein Ube3a Regulates Synaptic Growth and Endocytosis by Inhibiting BMP Signaling in *Drosophila*

**DOI:** 10.1371/journal.pgen.1006062

**Published:** 2016-05-27

**Authors:** Wenhua Li, Aiyu Yao, Hui Zhi, Kuldeep Kaur, Yong-chuan Zhu, Mingyue Jia, Hui Zhao, Qifu Wang, Shan Jin, Guoli Zhao, Zhi-Qi Xiong, Yong Q. Zhang

**Affiliations:** 1 Key Laboratory of Molecular and Developmental Biology, Institute of Genetics and Developmental Biology, Chinese Academy of Sciences, Beijing, China; 2 Institute of Neuroscience and State Key Laboratory of Neuroscience, Shanghai Institute of Biological Sciences, Chinese Academy of Sciences, Shanghai, China; 3 College of Life Science, Hubei University, Wuhan, Hubei, China; Harvard Medical School, UNITED STATES

## Abstract

Altered expression of the E3 ubiquitin ligase UBE3A, which is involved in protein degradation through the proteasome-mediated pathway, is associated with neurodevelopmental and behavioral defects observed in Angelman syndrome (AS) and autism. However, little is known about the neuronal function of UBE3A and the pathogenesis of UBE3A-associated disorders. To understand the *in vivo* function of UBE3A in the nervous system, we generated multiple mutations of *ube3a*, the *Drosophila* ortholog of *UBE3A*. We found a significantly increased number of total boutons and satellite boutons in conjunction with compromised endocytosis in the neuromuscular junctions (NMJs) of *ube3a* mutants compared to the wild type. Genetic and biochemical analysis showed upregulation of bone morphogenetic protein (BMP) signaling in the nervous system of *ube3a* mutants. An immunochemical study revealed a specific increase in the protein level of Thickveins (Tkv), a type I BMP receptor, but not other BMP receptors Wishful thinking (Wit) and Saxophone (Sax), in *ube3a* mutants. Ube3a was associated with and specifically ubiquitinated lysine 227 within the cytoplasmic tail of Tkv, and promoted its proteasomal degradation in Schneider 2 cells. Negative regulation of Tkv by Ube3a was conserved in mammalian cells. These results reveal a critical role for Ube3a in regulating NMJ synapse development by repressing BMP signaling. This study sheds new light onto the neuronal functions of UBE3A and provides novel perspectives for understanding the pathogenesis of UBE3A-associated disorders.

## Author Summary

Angelman syndrome (AS), characterized by severe mental retardation, developmental delay, ataxia, seizures, speech impairment, and happy disposition, is caused by mutation of E3 ubiquitin ligase UBE3A; a critical enzyme involved in proteasome-mediated protein degradation. Increasing evidence demonstrates that overexpression or hyperactivation of UBE3A is associated with autism. Thus, both loss and gain of UBE3A functions result in neurodevelopmental and cognitive defects. However, the neuronal functions of UBE3A and the mechanism by which altered expression of UBE3A leads to developmental and cognitive defects are poorly understood. Using *Drosophila melanogaster* as a model system in conjunction with an array of biochemical and physiological assays, we showed that mutants of *ube3a* had excess synaptic boutons and endocytic defects at the neuromuscular junction terminals due to an elevated level of bone morphogenetic protein (BMP) signaling. Specifically, Ube3a directly binds and ubiquitinates the BMP receptor Thickveins for proteasomal degradation; a function that is conserved in mammalian cells. Negative regulation of BMP signaling by UBE3A suggests a previously unknown molecular mechanism that underlies the pathogenesis of UBE3A-associated AS and autism.

## Introduction

Angelman syndrome (AS) is a neurodevelopmental disorder characterized by severe mental retardation, developmental delay, ataxia, seizures, speech impairment, and happy disposition [1, 2]. It is caused by disruption of the ubiquitin protein ligase E3A (UBE3A), which is expressed in the brain primarily from the maternal allele as a result of tissue-specific genomic imprinting [3, 4]. While loss of UBE3A causes AS, a maternal 15q11–13 duplication encompassing *UBE3A* results in autism [5–7]. A point mutation of UBE3A identified in an autism proband disrupts its phosphorylation by protein kinase A and increases its ligase activity [8]. Thus, both loss and gain of UBE3A function are associated with neurodevelopmental and cognitive defects.

UBE3A is involved in ubiquitin–proteasome-mediated protein degradation. Although several substrates of UBE3A have been identified, few have been implicated in neural development and function [9]. *Ube3a* mutant mice, a valid model of AS, show impairment in hippocampal long-term potentiation (LTP) [10, 11] and experience-dependent synaptic plasticity in the visual cortex [12, 13], together with imbalanced excitatory/inhibitory neurotransmission [14] and altered intrinsic membrane properties [15]. Moreover, Ube3a has been firmly implicated in the regulation of dendrites and spine development in neurons [16–18]. While the underpinnings of UBE3A-associated disorders remain to be elucidated, a recent study has shown that Ube3a targets the small-conductance potassium channel SK2 for degradation, and decreased NMDA receptor activation due to an elevated SK2 protein level underlies the impaired LTP in *Ube3a* mice [11]. Abnormal development and function of excitatory synapses in conjunction with a reduced number of AMPA receptors in *Ube3a* mutant mice are attributed to decreased Ube3a-mediated proteasomal degradation of RhoA guanine exchange factor (GEF) Ephexin5 and synaptic protein Arc [19, 20]. However, later studies showed that Arc is not a direct substrate of UBE3A [21, 22]. Thus, the molecular mechanisms by which UBE3A regulates synapse development and function remain unclear.

Null mutants of *ube3a*, the *Drosophila* homolog of human *UBE3A* [23], show defects in locomotive behavior, circadian rhythms, and long-term memory [24, 25], as well as reduced dendritic branching and growth of terminal dendritic processes of sensory neurons [26]. Similar to *ube3a* loss-of-function mutants, overexpression of wild-type *ube3a* also leads to motor abnormalities, circadian rhythm defects, and decreased dendritic branching [24–26]. So far, it has not been shown if *Drosophila ube3a* regulates synapse development. No direct targets of Ube3a-involved proteasomal degradation have been definitely identified in *Drosophila*, despite a series of proteomic studies [23, 27, 28].

Bone morphogenetic protein (BMP) pathway plays a crucial role in neural development throughout evolution [29–31]. In this study, we show that *Drosophila* Ube3a regulates synapse development and function at neuromuscular junctions (NMJs) by ubiquitinating and promoting proteasome-mediated degradation of the type I BMP receptor Tkv, and that the negative regulation of Tkv by Ube3a is conserved in mammalian cells. These results offer novel insight into the intricate regulation of BMP signaling in the nervous system, and suggest a potential intervention strategy for UBE3A-associated disorders by manipulating BMP signaling via genetic and pharmacological means.

## Results

### *ube3a* mutants are viable and fertile

**Fig 1:**
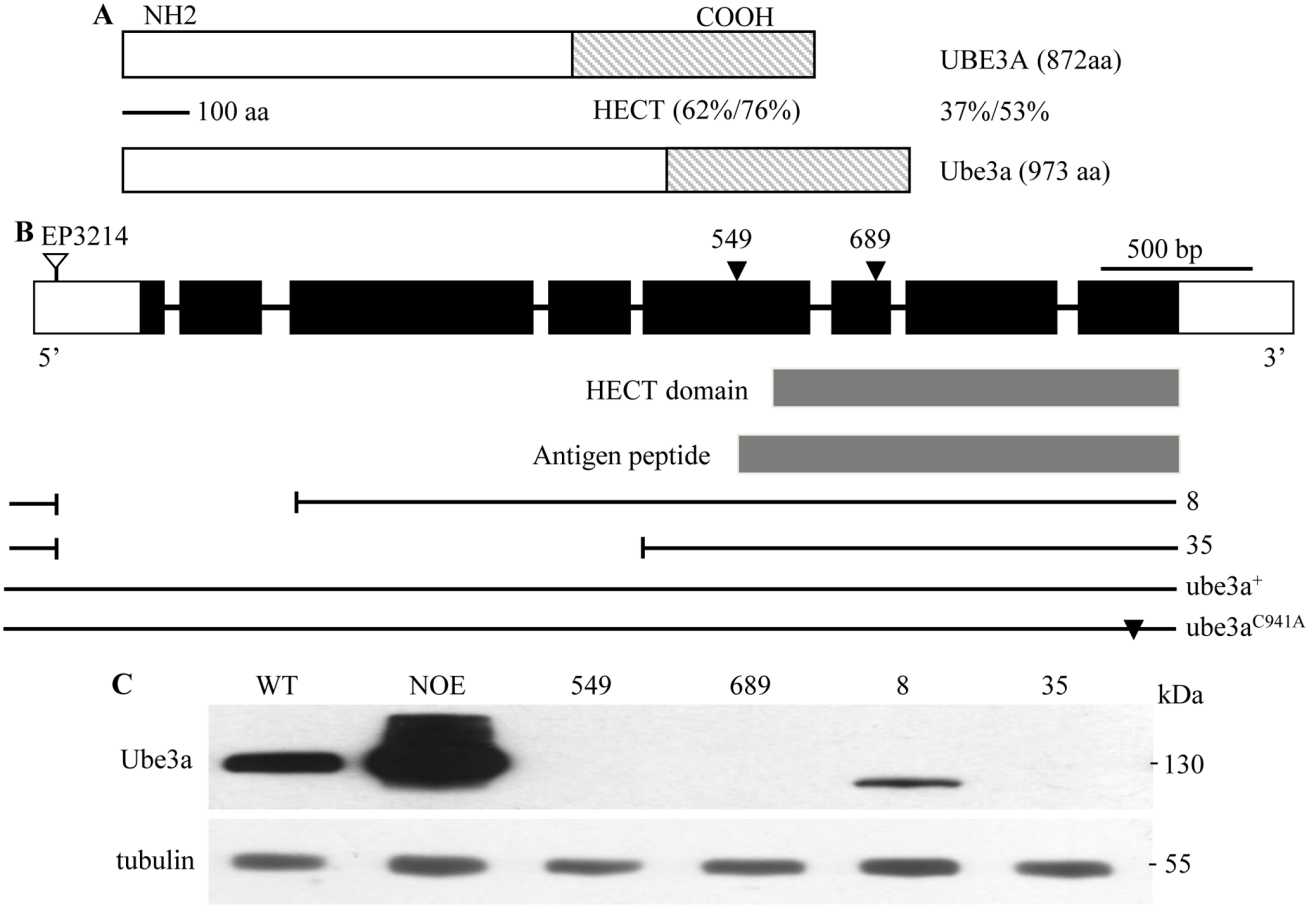
Characterization of *ube3a* mutations. (A) The percent amino acid identity/similarity between *Drosophila* Ube3a and human UBE3A. (B) Intron-exon organization and various mutations of *ube3a*. Coding and non-coding exons are represented by black and empty rectangles, respectively. Triangles indicate nonsense mutations; the HECT domain and the peptide used for antibody production are indicated. Two deletions, *ube3a*^*8*^ and *ube3a*^*35*^, produced by imprecise excision of the *P*-element insertion *EP3214* are depicted by interrupted lines. Genomic transgenes carrying wild-type (*ube3a*^*+*^) and mutant (*ube3a*^*C941A*^) containing a missense mutation in the codon encoding the catalytic cysteine residue *ube3a* are shown. (C) Western results of adult head extracts from various genotypes using the monoclonal antibody 8F7 against Ube3a. NOE denotes *elav-Gal4/UAS-ube3a*. No Ube3a expression was observed in hemizygous *ube3a*^*549*^ (*ube3a*^*549*^*/Df(3L)ED4470*) and *ube3a*^*689*^ (*ube3a*^*689*^*/Df(3L) ED4470*) mutants as well as homozygous *ube3a*^*35*^ mutants. A truncated Ube3a of ~110 kDa was observed in *ube3a*^*8*^ homozygous mutants. Tubulin was used as a loading control.

To examine the functions of Ube3a, we generated *ube3a* mutants through *P*-element-mediated excisions of *EP3214*, an insertion in the first exon. Multiple imprecise excisions (*e*.*g*., *ube3a*^*8*^ and *ube3a*^*35*^) were obtained (Fig 1B). In addition, two nonsense mutations (*ube3a*^*549*^ and *ube3a*^*689*^) induced by the chemical mutagen ethyl methanesulfonate (EMS) were identified via a TILLING service (see Materials and Methods) (Fig 1B). Western blotting using a mouse monoclonal antibody against the C-terminal peptide containing the HECT (Fig 1B) confirmed the absence of Ube3a protein in hemizygous *ube3a*^*549*^*/Df(3L)ED4470* (which removes the *ube3a* locus completely) and *ube3a*^*689*^*/Df(3L)ED4470* mutants and in homozygous *ube3a*^*35*^ mutants, whereas a truncated protein of 110 kDa was detected in *ube3a*^*8*^ mutants (Fig 1C). Immunostaining of larval and adult tissues using the monoclonal Ube3a antibody 8F7 showed that the endogenous Ube3a was cytoplasmic and widely expressed in various tissues including ventral ganglia, muscles, wing discs, ovaries and testes with an obvious enrichment at presynaptic NMJ terminals (S1 and S2 Figs). Both homozygous and hemizygous *ube3a*^*35*^ mutants, as well as independent null mutants generated in other laboratories [24, 26], were fully viable with no apparent developmental abnormalities, and both male and female adults were fertile.

### *ube3a* regulates NMJ development in presynaptic neurons

**Fig 2:**
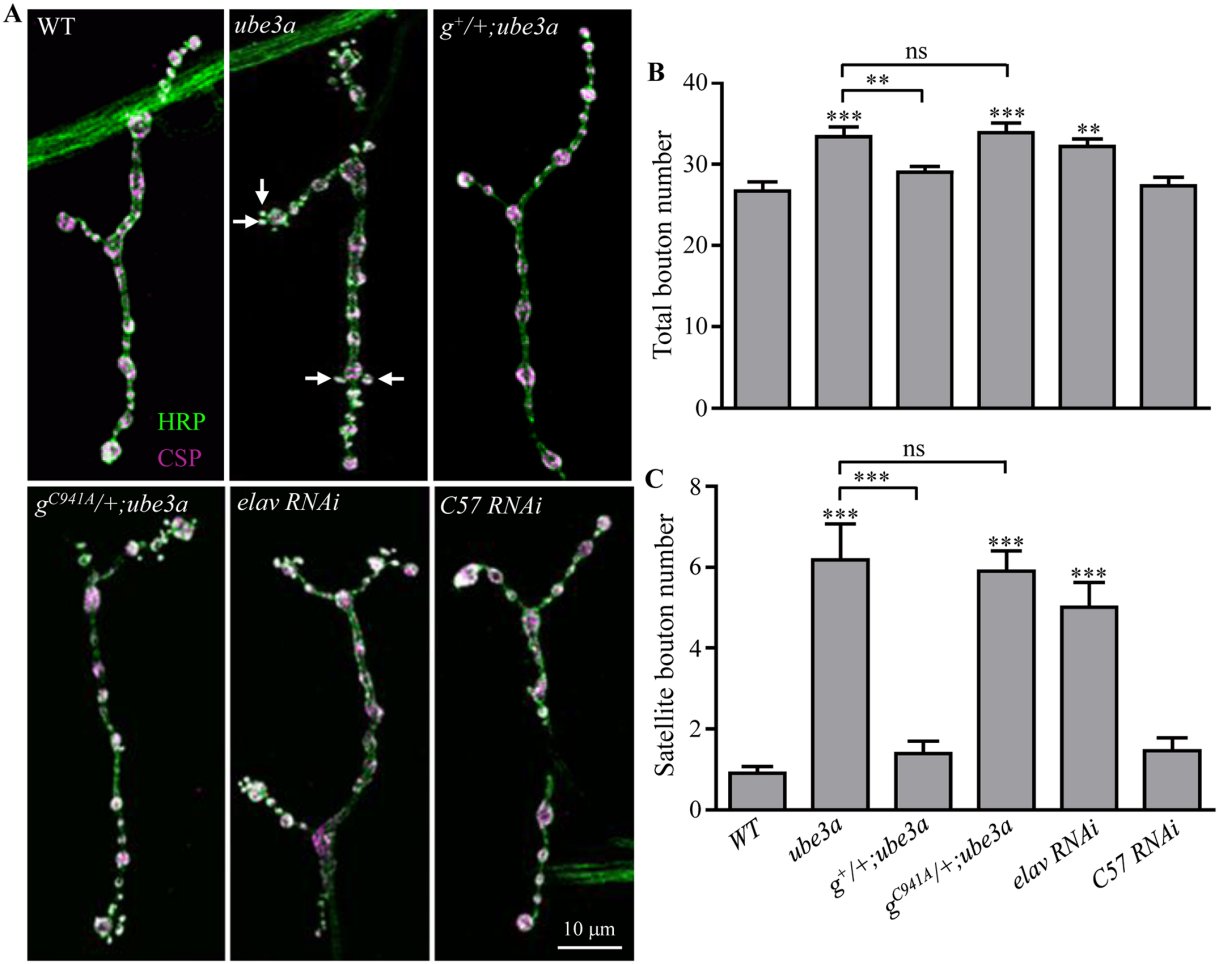
*ube3a* regulates NMJ synapse growth. (A) Muscle 4 NMJ synapses were double-stained with anti-HRP (green) and anti-CSP (magenta). The precise excision line *P95* was used as the wild-type control. *ube3a*^*35*^ homozygotes showed more total boutons and satellite boutons (indicated by arrows) than wild-type larvae. A copy of wild-type genomic *ube3a* transgene (*g*^*+*^) rescued the NMJ phenotype of *ube3a*^*35*^ mutants, however, a copy of *ube3a* transgene harboring the missense mutation C941A (*g*^*C941A*^) did not. *elav-Gal4*-driven *Thu3266* RNAi against *ube3a* in presynaptic neurons led to increased numbers of total and satellite boutons, while *C57-Gal4*-driven postsynaptic knockdown of Ube3a had no effect on NMJ growth. Scale bar = 10 μm. (B, C) Quantification of total bouton (B) and satellite bouton (C) number in different genotypes including wild type, *ube3a*^*35*^, genomic *ube3a*^*+*^*/+*; *ube3a*^*35*^, genomic *ube3a*^*C941A*^*/+*; *ube3a*^*35*^, *elav-Gal4/+*; *RNAi/+*, and *RNAi/+*; *C57-Gal4/+*. *n* ≥ 18 NMJs; **P < 0.01, ***P < 0.001; ns, not significant. Error bars represent SEM.

Given the synaptic localization of Ube3a in cultured hippocampal neurons [16], the impaired LTP and defective synaptic transmission and plasticity observed in *Ube3a* mutant mice [10, 11, 14], and the presynaptic enrichment of Ube3a (S1 Fig), we hypothesized that *Drosophila ube3a* may play an important role at synapses. To test this possibility, we examined the NMJs of wandering third instar larvae by staining them with anti-cysteine string protein (CSP), a synaptic vesicle marker, and anti-horseradish peroxidase (HRP), which labels the presynaptic neuronal membrane. In *ube3a*^*35*^ null mutants, the total bouton number for muscle 4 NMJs in abdominal segments A2 and A3 was 33.4 ± 1.2, which was significantly higher than 26.8 ± 1.1 in wild-type (WT) larvae (*P* < 0.001) (Fig 2A and 2B). Similar to homozygous *ube3a*^*35*^ mutants, allelic combination of *ube3a*^*35*^*/ube3a*^*549*^ and hemizygous *ube3a*^*35*^*/Df(3L)ED4470* and *ube3a*^*549*^*/Df(3L)ED4470* also showed more boutons than the wild type. The number of satellite boutons budding from a parental bouton in *ube3a*^*35*^ mutants was also significantly higher than in wild-type larvae; these satellites were more frequent at the distal ends of NMJ terminals (Fig 2A and 2C). To confirm that the observed phenotype was attributable to loss of *ube3a*, we conducted rescue experiments using a genomic transgene. One copy of wild-type *ube3a* restored the number of total and satellite boutons in *ube3a*^*35*^ mutants (Fig 2A, 2B and 2C). However, a *ube3a* genomic transgene carrying the missense mutation C941A disrupting the thioester bond with ubiquitin did not (Fig 2A, 2B and 2C), although it was expressed at a level comparable to the wild-type genomic transgene.

To determine whether *ube3a* exerts its effect on the pre- or postsynaptic side, we knocked down Ube3a expression by RNA interference (RNAi) in a tissue-specific manner. Targeted knockdown of *ube3a* in presynaptic neurons by *elav-Gal4*-driven *Thu3266* RNAi resulted in a modest but significant increase in the number of total boutons (20.15%) and a dramatic increase in satellite boutons (371.7%) compared to wild-type larvae (Fig 2). Motoneuron specific knockdown of *ube3a* by *OK6-Gal4* also showed overgrown NMJs (S3 Fig), whereas knockdown of *ube3a* in postsynaptic muscles using *C57-Gal4* had no effect on bouton number (*P* > 0.05 compared to wild type; Fig 2). Similar results were observed using an independent RNAi line *VDRC45876*. These findings together demonstrate that Ube3a regulates NMJ synapse growth presynaptically.

### Synaptic endocytosis is impaired in *ube3a* mutants

An increased number of satellite boutons at NMJs is closely associated with a defect in endocytosis [32–34]. To find out if *ube3a* mutant NMJs exhibited an endocytic defect, we first examined NMJ synapses by electron microscopy. The critical synaptic structures such as active zones and subsynaptic reticulum appeared normal in *ube3a* mutant boutons. Specifically, the mean density of synaptic vesicles (SVs) within a 200 nm radius of the active zone of transmitter release was normal in *ube3a* mutants compared to wild type (S4 Fig), as reported previously [35]. However, significantly more enlarged vesicles > 60 nm in diameter (also known as cisternae, presumably endosomes) per bouton cross-section were observed in *ube3a* mutants (5.06 ± 0.90 in *ube3a* vs 1.19 ± 0.21 in wild type; *n* ≥ 38 boutons from ≥ 4 larvae, ***p < 0.001; S4 Fig), suggesting a vesicle formation defect.

**Fig 3:**
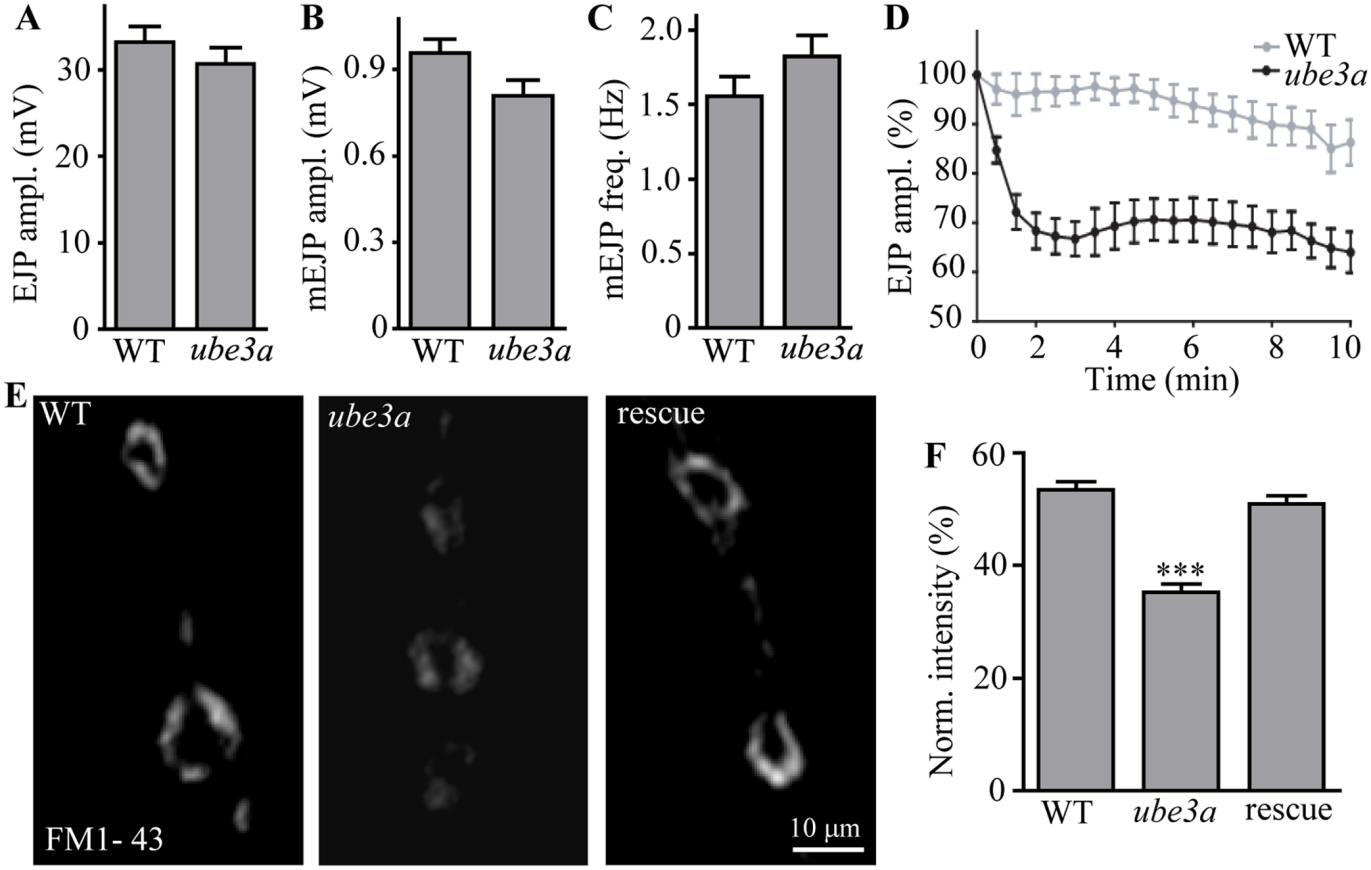
Synaptic endocytosis is impaired in *ube3a* mutants. (A–C) Statistical results of EJP amplitudes (A), mEJP amplitudes (B), and mEJP frequencies (C) of wild type and *ube3a*^*35*^ mutants. (D) Relative EJP amplitudes (%) during high-frequency stimulation (10 Hz) for 10 min in wild type and *ube3a*^*35*^ mutants. *n* ≥ 11 larvae; error bars indicate SEM. (E) Loss of *ube3a* causes synaptic endocytic defects. FM1-43 dye uptake in wild type, *ube3a*^*35*^ mutants, and rescued animals (*gube3a*^*+*^*/+; ube3a*^*35*^). Scale bar: 10 μm. (F) Normalized fluorescence intensity (%) of endocytosed FM1-43 for wild type, *ube3a*^*35*^ and rescued mutants (*gube3a*^*+*^*/+; ube3a*^*35*^). *n* ≥ 20 NMJs, error bars indicate SEM, *** indicates *p* < 0.001.

We then examined both evoked and spontaneous synaptic glutamate release at *ube3a*^*35*^ mutant NMJs using intracellular recordings. We first stimulated motor neurons at a low frequency of 0.3 Hz in the presence of 0.5 mM Ca^2+^. The amplitudes of excitatory junctional potentials (EJPs) and miniature EJPs (mEJPs) in *ube3a*^*35*^ mutants did not differ from those in the controls (Fig 3A and 3B). The mEJP frequency in *ube3a* mutants was similar to that in wild-type larvae (Fig 3C). Representative traces of EJP and mEJP of wild type (A) and *ube3a*^*35*^ mutant (B) are shown in S5 Fig. Thus, the basal transmission appeared normal in *ube3a* mutants. We then examined the ability of *ube3a* mutants to maintain neurotransmitter release during intense stimulation. The EJP amplitudes in *ube3a* mutants under high-frequency stimulation of 10 Hz in the presence of 0.5 mM extracellular Ca^2+^ for 10 min declined much faster and remained at 64% of the initial response (Fig 3). In contrast, control animals sustained release at about 85% of the initial amplitude of EJP (Fig 3D). The inability of *ube3a* mutants to maintain normal levels of transmission during intense activity is consistent with a specific defect in vesicle trafficking or recycling.

Finally, to examine whether endocytosis is affected in *ube3a* mutant NMJ synapses, we performed fluorescent dye FM1-43 uptake experiments. There was intense labeling of FM1-43 at wild-type synaptic boutons, whereas *ube3a*^*35*^ mutants showed significantly reduced uptake of FM1-43 by 34.1% compared to wild type (*p* < 0.001; Fig 3E and 3F). Importantly, the endocytosis defect could be fully rescued by one copy of the genomic *ube3a* transgene (Fig 3E and 3F). The failure to maintain neurotransmission under tetanic stimulation and reduced dye uptake support that *ube3a* is required for efficient synaptic vesicle endocytosis.

### *ube3a* regulates NMJ growth through the BMP signaling pathway

*ube3a* mutations resulted in more satellite boutons and endocytic defects (Figs 2 and 3). We investigated the underlying mechanism of the synaptic defects in *ube3a* mutants. Synapse formation requires the coordinated activity of several signaling cascades. At the *Drosophila* NMJ, the BMP pathway acts in a retrograde manner from muscles to motor neurons and promotes NMJ growth [30, 34, 36–39]. We hypothesized that the overgrowth of synaptic boutons in *ube3a* mutants may be caused by elevated BMP signaling. To test this possibility, we examined genetic interactions between *ube3a* and the major genes of the BMP signaling pathway, *tkv* and *mad*.

**Fig 4:**
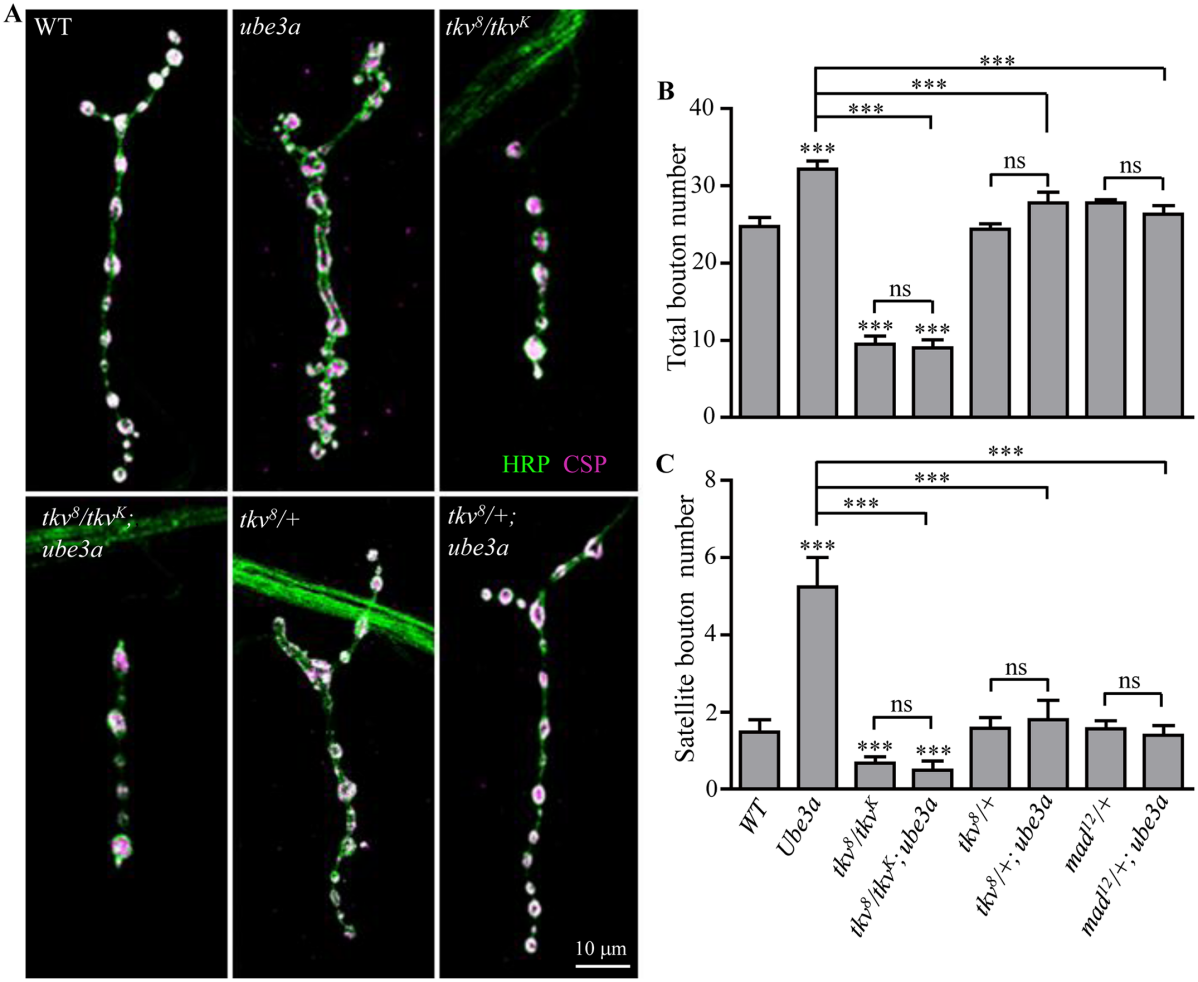
*ube3a* interacts genetically with BMP signaling pathway components *tkv* and *mad* in regulating NMJ growth. (A) Confocal images of muscle 4 NMJ synapses co-stained with anti-CSP (magenta) and anti-HRP (green) to reveal synaptic vesicles and presynaptic membrane, respectively. In *ube3a tkv* double mutants, bouton numbers were similar to those of *tkv*^*8*^*/tkv*^*K16713*^ single mutants. One copy of *tkv*^*8*^ rescued the synapse overgrowth in *ube3a*^*35*^ mutants. Scale bar = 10 μm. (B, C) Quantification of total bouton number (B) and satellite bouton number (C) of various genotypes including wild type, *ube3a*^*35*^, *tkv*^*8*^*/tkv*^*K16713*^, *tkv*^*8*^*/ tkv*^*K16713*^; *ube3a*^*35*^, *tkv*^*8*^*/+*, *tkv*^*8*^*/+; ube3a*^*35*^, *mad*^*12*^*/+*, *mad*^*12*^*/+; ube3a*^*35*^. *n* ≥ 18 NMJ terminals, one-way ANOVA test, ***P < 0.001, ns, not significant,. Error bars represent SEM.

Compared to wild type, muscle 4 NMJ terminals were overgrown in *ube3a*^*35*^ mutants as described above, but severely underdeveloped in *tkv*^*8*^*/tkv*^*K16713*^ mutants (Fig 4A and 4B). In *ube3a tkv* double mutants, bouton numbers were similar to that of *tkv* single mutants (Fig 4A and 4B), suggesting that the overgrowth of NMJ synapses produced by loss of *ube3a* may be mediated by alterations in BMP signaling. Furthermore, NMJ overgrowth in *ube3a* mutants was dependent on the level of Tkv and the BMP effector Mad. Loss of one copy of *tkv*, which alone had no effect on bouton number, reversed the increase in satellite bouton number caused by loss of Ube3a (Fig 4A, 4B and 4C). Mutating one copy of *mad* also suppressed the overgrowth of NMJ synapses in *ube3a* mutants (Fig 4B and 4C). These results indicate that the excessive synaptic growth induced by loss of *ube3a* is mediated by elevated levels of the BMP signaling components Tkv, Mad, or both.

### Increased BMP signaling in *ube3a* mutants

**Fig 5:**
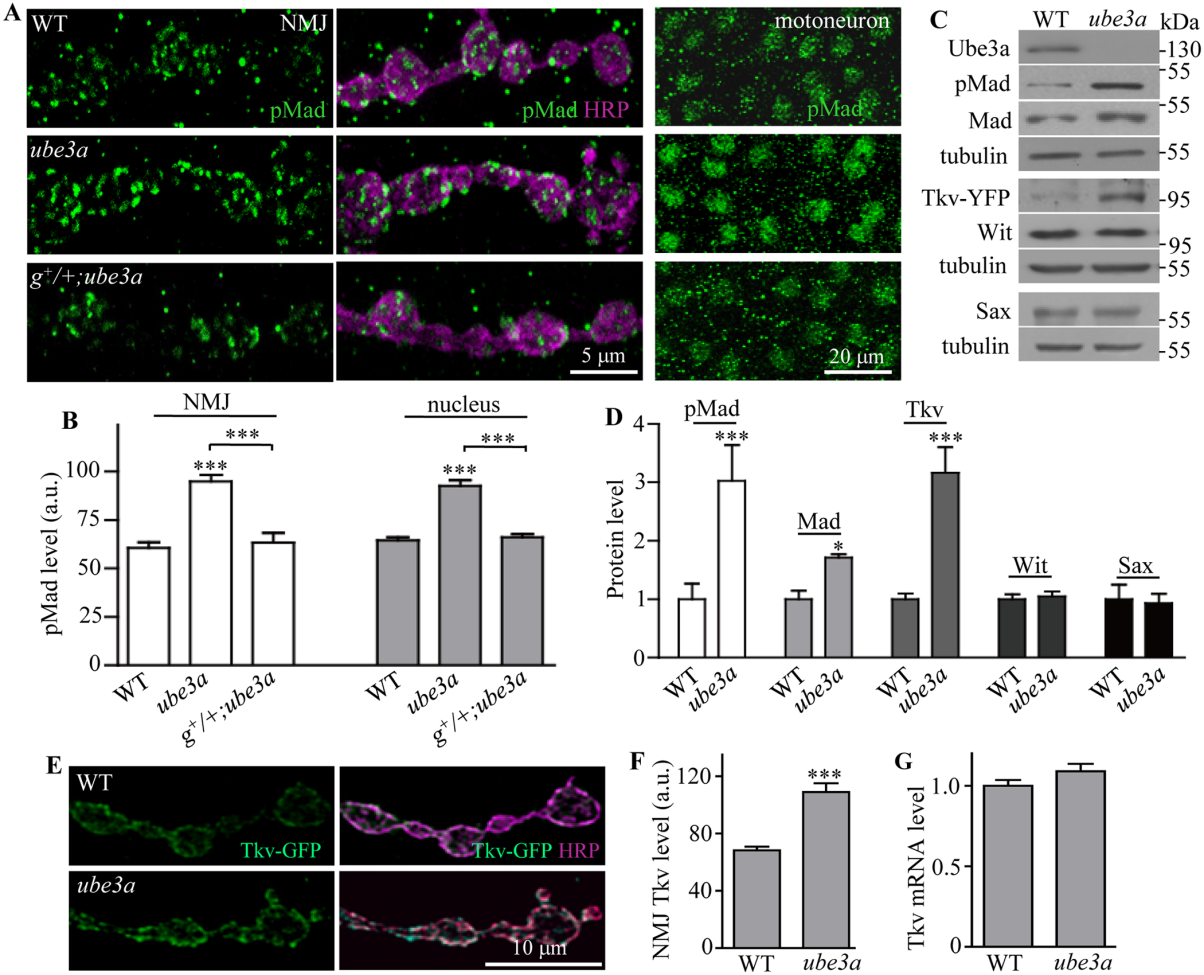
Levels of pMad and Tkv protein are increased in *ube3a* mutants. (A) Increased level of pMad at the NMJ synapses and in the motoneuron nuclei of *ube3a*^*35*^ mutants was rescued by one copy of the wild-type genomic *ube3a* transgene (*g*^*+*^). (B) Statistical analysis of mean fluorescence intensities in arbitrary units (a.u.) for pMad in NMJs and motoneuron nuclei of different genotypes (*n* = 4, one-way ANOVA test, mean ± SEM, ***P < 0.001). (C) Increased levels of pMad, Mad, and Tkv-YFP, but not the endogenous Wit and Sax protein, in larval brains of *ube3a*^*35*^ mutants. Tkv protein level was examined from a gene trap line expressing the endogenous Tkv tagged with YFP. α-Tubulin was used as a loading control. (D) Statistical analysis of different protein levels in *ube3a*^*35*^ mutant brains (*n* = 4, *t* test, mean ± SEM, *P < 0.05, ***P < 0.001). (E) An increased level of *elav-Gal4*-driven Tkv-GFP in *ube3a*^*35*^ mutant NMJs compared with wild type. Scale bar = 10 μm. (F) Quantitative analysis of Tkv-GFP fluorescence intensities in a.u. normalized to HRP-positive area at NMJs of wild-type and *ube3a*^*35*^ mutant larvae (*n* = 4, *t* test, mean ± SEM, ***P < 0.001). (G) Normal level of *tkv* mRNA in *ube3a*^*35*^ mutant larval brains examined by quantitative PCR. *tkv* mRNA level was normalized to *actin* mRNA (*n* = 3, *t* test, mean ± SEM).

BMP receptor activation leads to phosphorylation of Mad (pMad), which then translocates to the nucleus to activate target gene transcription. Elevated pMad levels are thus indicative of active BMP signaling [34, 40, 41]. To determine if *ube3a* loss-of-function does indeed lead to NMJ overgrowth through upregulated BMP signaling, we quantified the level of pMad in NMJ synapses and motoneuron nuclei. pMad staining intensity at NMJ synapses was significantly increased by 56.5% in *ube3a*^*35*^ mutants compared to wild type (*P* < 0.001; Fig 5A and 5B). The intensity of pMad immunofluorescence was also significantly higher in motoneuron nuclei of *ube3a* mutants than in wild types (*P* < 0.001; Fig 5A and 5B). Furthermore, pMad expression was elevated in larval brain extracts as measured by western analysis (Fig 5C and 5D). One copy of the genomic *ube3a* transgene fully reversed the increased pMad levels at both NMJs and motoneuron nuclei of *ube3a* mutants (Fig 5A and 5B). These results show that *ube3a* negatively regulates pMad expression at both NMJs and motoneuron nuclei. Remarkably, the increased pMad staining, together with reduced dye loading and faster rundown of EJP amplitudes, in *ube3a* mutants is fully rescued by reducing the dose of Tkv by half (S6 Fig).

Given that Ube3a is an E3 ubiquitin ligase, we examined the protein expression levels of BMP signaling components in *ube3a* mutants to determine whether any are Ube3a substrates. Western blotting of larval brain extracts showed that pMad level was significantly increased by 203%, while Mad level was increased by 71% in *ube3a* mutants, consistent with the staining results (Fig 5A–5D). As a high quality of antibody against Tkv was not readily available, we took advantage of a yellow fluorescent protein (YFP) gene trap line that expresses functional YFP-tagged Tkv (Tkv-YFP) in the ventral nerve cord, including motoneurons under the control of the endogenous promoter (S7 Fig). Western blotting with an anti-GFP antibody that also recognizes YFP, showed that the protein level of Tkv-YFP was increased by 216% in *ube3a* mutants compared to wild type (*P* < 0.001), while the level of the endogenous Sax and Wit, the other type I and type II BMP receptors, respectively, was unaltered (Fig 5C and 5D). This suggests that Tkv is a primary target of Ube3a-mediated degradation. Indeed, the fluorescence intensity of *elav-Gal4*-driven Tkv-GFP was elevated by 59.2% at NMJ synapses of *ube3a* mutants compared to wild type (Fig 5E and 5F). Quantitative real-time PCR, however, detected similar levels of *tkv* mRNA in the brains of *ube3a*^*35*^ mutant and wild-type larvae (Fig 5G), indicating that the increase in Tkv protein expression in *ube3a* mutants is likely due to post-transcriptional regulation.

**Fig 6:**
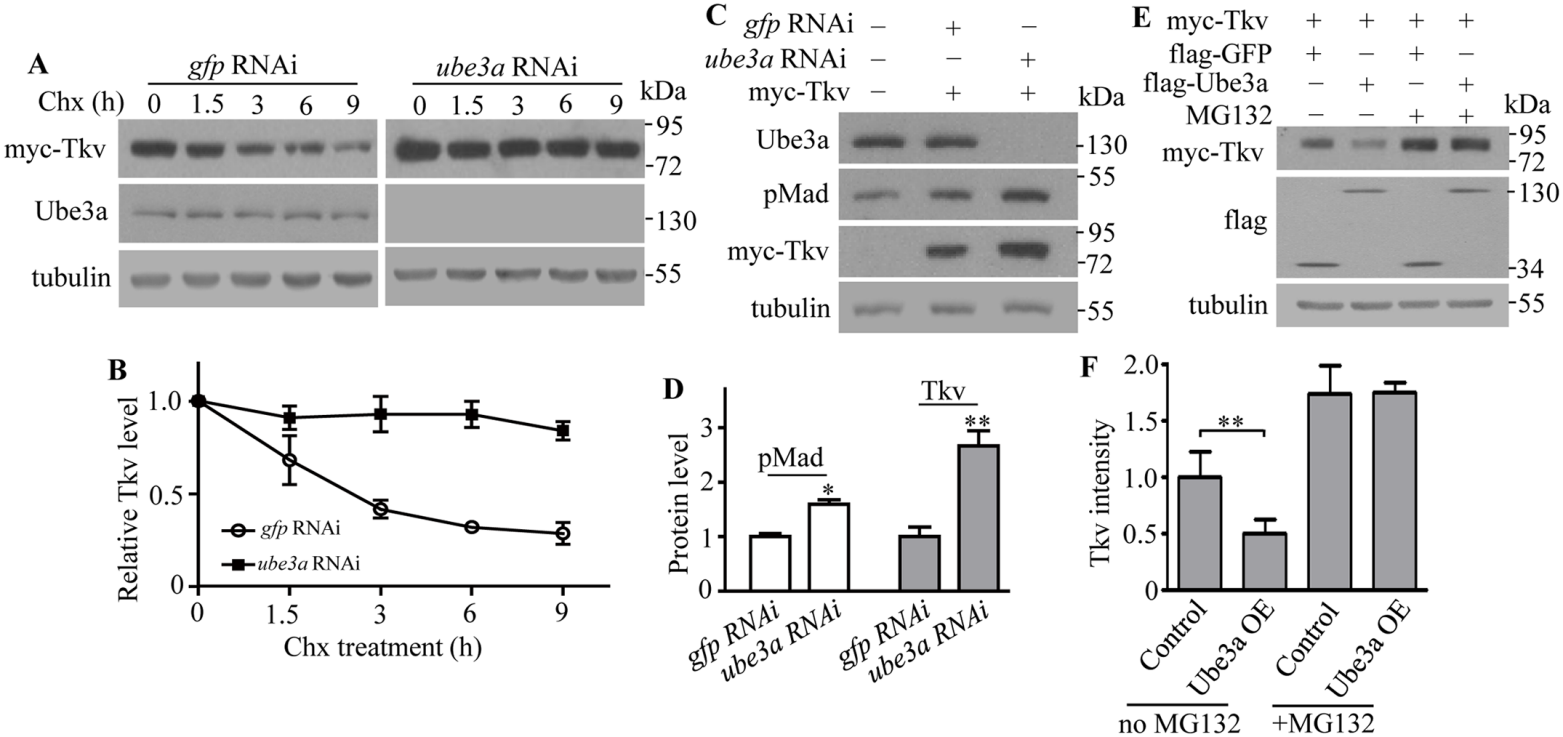
Ube3a negatively regulates Tkv protein levels in S2 cells. (A) Tkv protein levels in S2 cells treated with dsRNAs targeting *ube3a* or *gfp* (control) at various times after blocking protein synthesis by cycloheximide treatment. (B) A slowed decline in Tkv protein levels in S2 cells treated with cycloheximide after knockdown of *ube3a* by dsRNA. Values are shown as the ratio of Tkv intensity to tubulin control, normalized to the untreated cells at time zero (*n* = 3, mean ± SEM). (C) An increase in Tkv protein level after Ube3a knockdown in S2 cells. dsRNA against GFP was used as a control. (D) Statistical analysis of pMad and Tkv protein levels in S2 cells expressing a reduced level of Ube3a by RNAi (*n* = 4, one-way ANOVA test, mean ± SEM, *P < 0.05, **P < 0.01). (E) A decrease in Tkv protein level was observed in Ube3a-overexpressing S2 cells; the effect was abolished by treatment with the 26S proteasome inhibitor MG132. The experiment was repeated three times with similar results. (F) Statistical analysis of relative Tkv protein level normalized to the loading control tubulin in S2 cells co-expressing Ube3a with or without MG132 treatment (*n* = 3, one-way ANOVA test, **P < 0.01, mean ± SEM).

As Tkv acts upstream of Mad, and Tkv protein level is obviously upregulated in *ube3a* mutants, we focused on the mechanisms mediating negative regulation of Tkv by Ube3a by examining the stability of Tkv protein in S2 cells at various time points after treatment with the protein synthesis inhibitor cycloheximide. In control cells transfected with double-stranded RNAs (dsRNAs) against GFP, Tkv level decreased markedly from 1.5 h to 9 h upon drug treatment (Fig 6A). In Ube3a knockdown cells, however, a relatively stable level of Tkv was observed (Fig 6A and 6B). Ube3a knockdown resulted in a significantly elevated level of pMad (1.6 times the control) and Tkv (2.7 times the control) at 48 h after dsRNA transfection (Fig 6C and 6D). This effect was confirmed by using a distinct dsRNA targeting a different sequence of *ube3a* mRNA, demonstrating the specificity of Tkv downregulation by Ube3a.

To determine whether Tkv stability is regulated by the proteasomal degradation pathway, Tkv protein level was measured in Ube3a-overexpressing S2 cells treated with the 26S proteasome inhibitor MG132. In contrast to the upregulation of Tkv observed upon Ube3a knockdown, Tkv levels were significantly reduced by 50.17% in Ube3a-overexpressing cells compared with GFP-overexpressing control cells, and this effect was abolished by MG132 treatment (Fig 6E). These results indicate that Ube3a negatively regulates Tkv protein levels by targeting the protein for proteasome-mediated degradation.

### Tkv is a direct substrate of Ube3a

**Fig 7:**
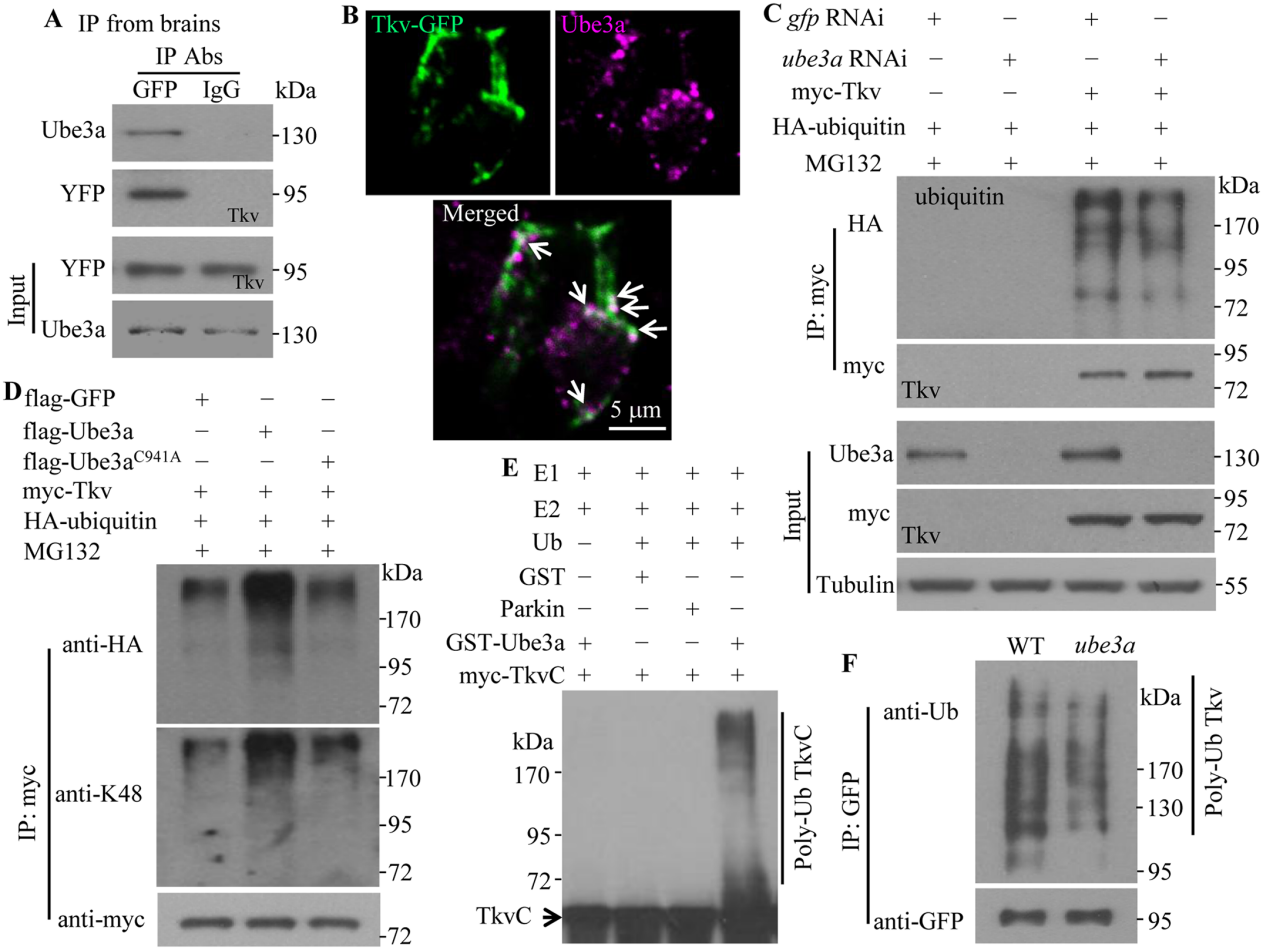
Ube3a interacts with and ubiquitinates Tkv *in vivo* and *in vitro*. (A) Ube3a interacts with Tkv in larval brains as detected by co-IP. Ube3a was co-immunoprecipitated by anti-GFP from larval brain lysates of a gene trap line expressing endogenous Tkv tagged with YFP. (B) Co-localization of Tkv-GFP and Ube3a driven by *elav-Gal4* in motoneuron soma. Images from two consecutive single slices are presented. Arrows indicate co-localized puncta. (C) Decreased Tkv ubiquitination by RNAi knockdown of Ube3a in S2 cells. Cells were co-transfected with plasmids encoding myc-tagged Tkv and HA-tagged ubiquitin, and treated with dsRNA against Ube3a or GFP dsRNA as a control. Anti-HA antibody was used to detect ubiquitinated Tkv. Tubulin was used as a loading control. (D) Increased Tkv poly-ubiquitination by overexpression of wild-type but not C941A mutant Ube3a in S2 cells. Poly-ubiquitination was detected using anti-HA and anti-K48 antibodies. (E) GST-Ube3a ubiquitinates myc-TkvC *in vitro*. The reaction was reconstituted with the components E1, E2, HA-Ub, the potential substrate myc-TkvC, together with the GST control, the E3 ligase Parkin control, or GST-Ube3a. Both poly-ubiquitinated and non-ubiquitinated TkvC are indicated. (F) *In vivo* ubiquitination of Tkv. Tkv-GFP from larval brain lysates was immunoprecipitated with anti-GFP and the level of ubiquitinated Tkv was detected by anti-ubiquitin. A decreased level of Tkv ubiquitination was observed in *ube3a*^35^ mutants compared with wild type.

Given the increased stability of Tkv in Ube3a knockdown cells and the involvement of the proteasome in Tkv degradation (Fig 6), Tkv may be a direct target for Ube3a-mediated ubiquitination and ensuing proteasome-mediated degradation. This model predicts a physical interaction between the two, and direct ubiquitination of Tkv by Ube3a. We investigated the physical interaction between Ube3a and Tkv by co-immunoprecipitation (co-IP). In third instar larval brain extracts, endogenous Ube3a co-precipitated with Tkv-YFP using an antibody against GFP, but was absent in control immunoprecipitates using anti-IgG (Fig 7A). We defined the regions mediating the interaction in S2 cells and found that the N-terminal 201–400 and 400–640 amino acid fragments of Ube3a each interacted with Tkv, while the STYKc domain of Tkv mediated its interaction with Ube3a (S8 Fig). Co-localization of Tkv-GFP and Ube3a driven by *elav-Gal4* was observed in the soma of motoneurons in the ventral ganglion (6.3 puncta positive for both Tkv-GFP and Ube3a per neuron, *n* = 16; Fig 7B), further supporting a physical interaction between Ube3a and Tkv.

To establish whether Tkv is a direct substrate of Ube3a-mediated ubiquitination, we measured the level of unmodified and ubiquitinated Tkv in S2 cells. In Ube3a knockdown cells, Tkv poly-ubiquitination was reduced (Fig 7C). Conversely, overexpression of Ube3a caused an increase in Tkv poly-ubiquitination (Fig 7D). Poly-ubiquitination occurred at lysine 48 of ubiquitin (Fig 7D); a form of poly-ubiquitination that targets proteins for proteasomal degradation [42]. An *in vitro* ubiquitination assay further revealed that GST-Ube3a induced poly-ubiquitination of myc-TkvC, while neither GST alone, the control E3 ligase Parkin, nor a cocktail missing the essential component ubiquitin, led to TkvC poly-ubiquitination (Fig 7E). To verify if the negative of BMP signaling by Ube3a occurred *in vivo*, we treated dissected larvae with MG132 for 4 h and observed a significantly increased level of pMad at both NMJs and motoneuron nuclei (S9 Fig). Blocking proteasome function by expressing a dominant negative proteasomal subunit DTS5 in presynaptic neurons by *elav-Gal4* also resulted in a similar increase of pMad staining (S9 Fig). Western blotting of larval brain lysates showed a decreased level of ubiquitinated Tkv in *ube3a* mutants (Fig 7F). These results demonstrate that Ube3a promotes ubiquitin-mediated degradation of Tkv.

**Fig 8:**
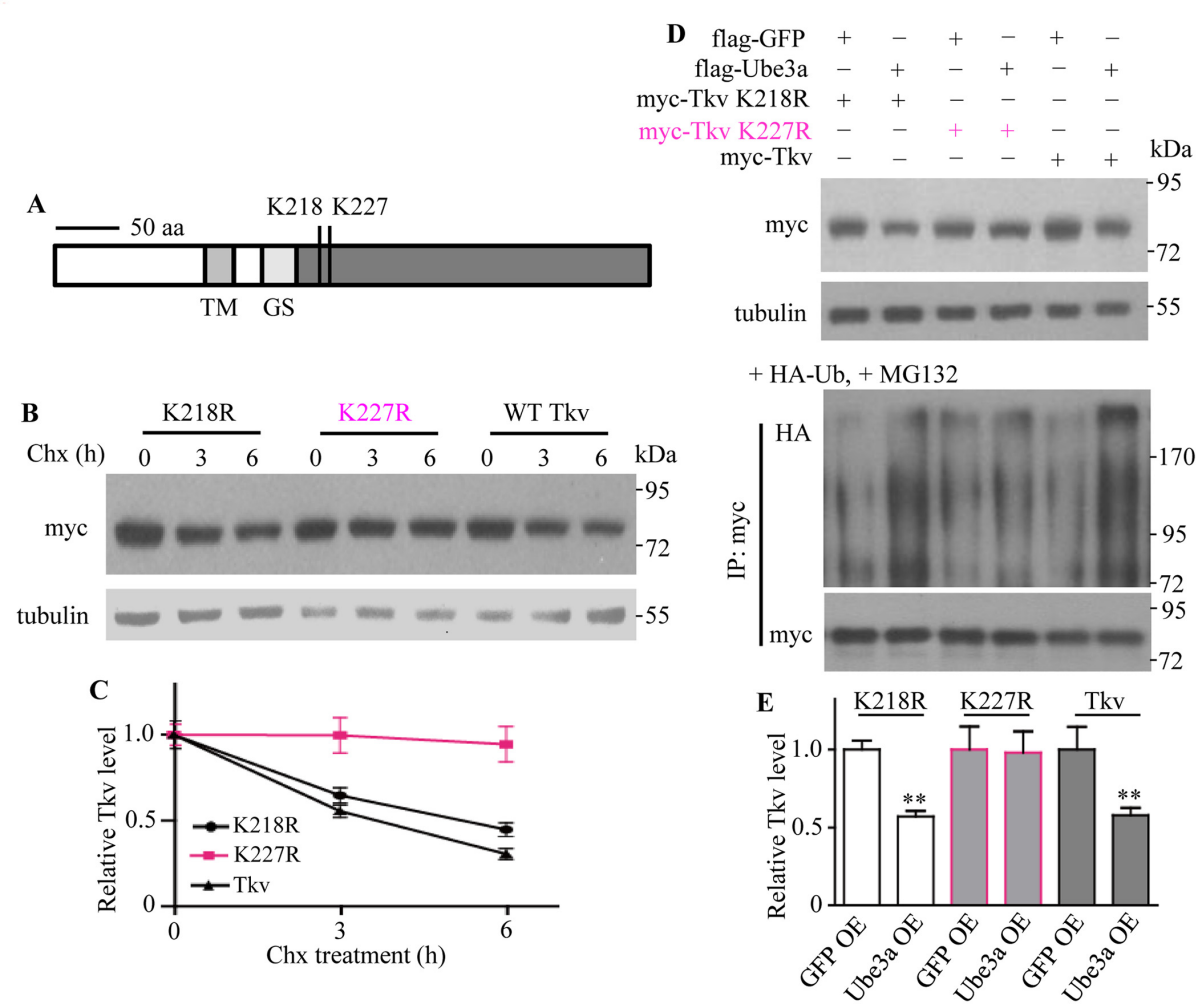
Identification of Tkv ubiquitination sites targeted by Ube3a. (A) Lysine K218 and K227 in the cytoplasmic region of Tkv were identified as ubiquitination sites by mass spectrometry. Scale bar indicates 50 amino acids. TM indicated transmembrane domain; GS denotes Gly-Ser rich motif. (B) K227R Tkv was more stable than K218R or wild-type Tkv. S2 cells transfected with Ube3a and wild-type or mutant Tkv were treated with cycloheximide to block protein synthesis, and the level of myc-Tkv was detected to determine protein stability. (C) Statistical analysis of wild-type and mutant Tkv protein levels at different time points after protein synthesis blockade by cycloheximide (*n* = 3, mean ± SEM). (D) Verification of Tkv ubiquitination sites targeted by Ube3a. S2 cells were transfected with plasmids encoding Ube3a and wild-type or mutant Tkv. After 48 h transfection, the level of Tkv was examined by western blotting (upper blot). For ubiquitination assays, S2 cells over-expressing Ube3a and wild-type or mutant Tkv, together with HA-ubiquitin (+HA-Ub) and treated with MG132 (+MG132) to block ubiquitin-mediated degradation were immunoprecipitated with anti-myc antibody. Western blotting was performed to analyze the levels of ubiquitinated and un-ubiquitinated Tkv. (E) Statistical analysis of wild-type and mutant Tkv protein levels normalized to the loading control tubulin when co-expressed with Ube3a in S2 cells (*n* = 3, one-way ANOVA test, **P < 0.01, mean ± SEM).

Given that Ube3a ubiquitinated Tkv and promoted its degradation by proteasome pathway (Figs 6 and 7), we aimed to identify specific amino acids of Tkv targeted by UBE3A. Mass spectrometry revealed two potential ubiquitinated residues, lysine 218 and 227, in the C-terminal STYKc region of Tkv (Fig 8A). To verify which specific sites of Tkv were ubiquitinated by Ube3a, we mutated lysine to arginine at two individual sites, namely, K218R and K227R. We compared the stability of wild-type and mutant Tkv protein in S2 cells by western blotting following treatment with cycloheximide to inhibit protein synthesis for 0, 3 and 6 h. We found that the stability of K218R was comparable to that of wild-type Tkv, the levels of both decreased with time, while the amount of K227R remained stable within 6 h treatment of cycloheximide (Fig 8B and 8C). Ubiquitination analysis showed that K218R was as sensitive as wild-type Tkv for ubiquitination in S2 cells by Ube3a in the presence of HA-Ub and MG132 (Fig 8D and 8E). There was a concomitant significant reduction in wild-type and mutant Tkv protein level, while K227R was stable and ubiquitination resistant (Fig 8D and 8E). These results demonstrate that K227 is a key target residue for ubiquitination by Ube3a.

### Human UBE3A binds and ubiquitinates ALK3, a mammalian homolog of Tkv

**Fig 9:**
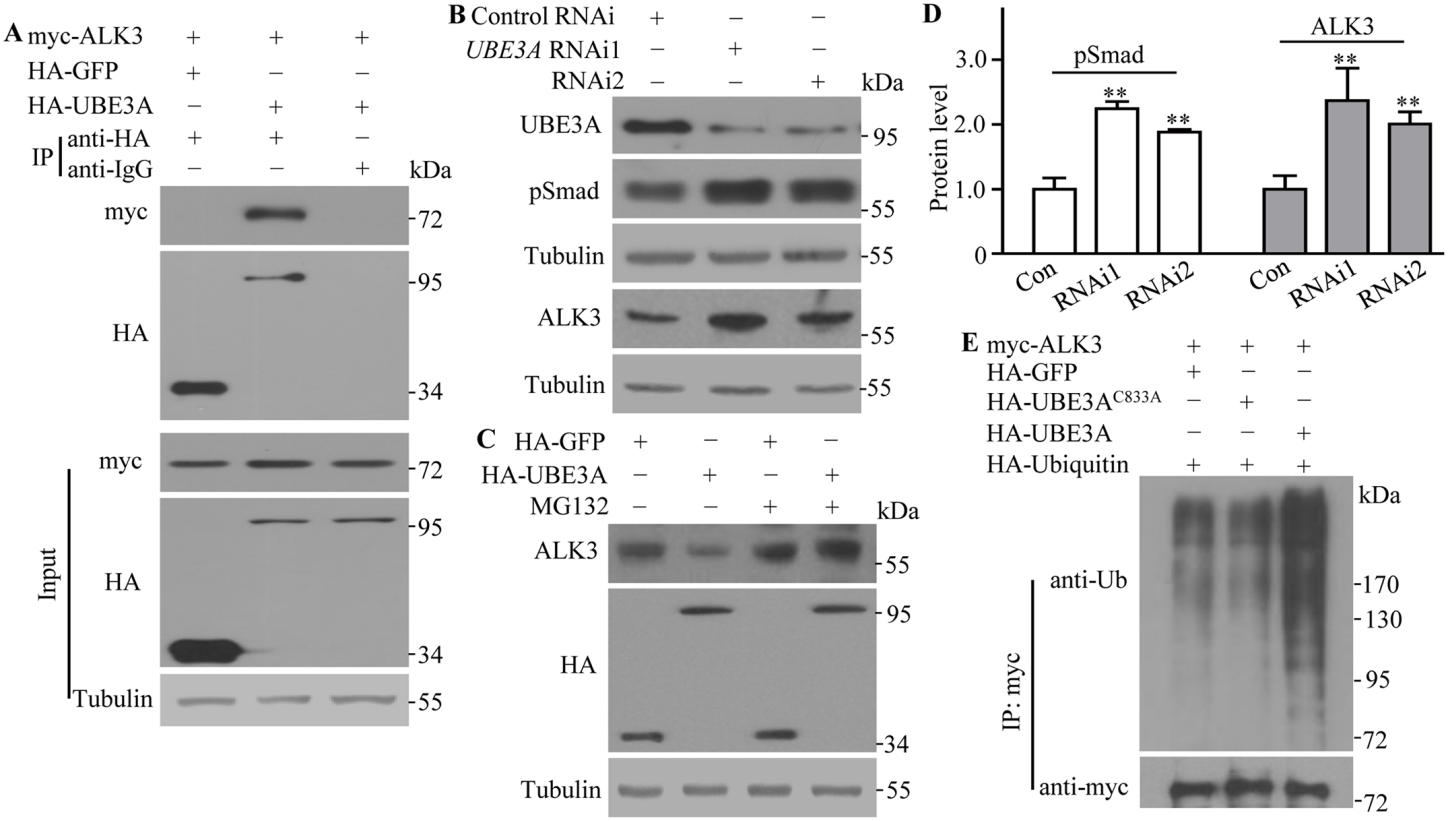
Human UBE3A negatively regulates the protein level of BMP receptor ALK3 in HEK293 cells. (A) Human UBE3A interacts with the BMP receptor ALK3 in HEK293 cells. HEK293 cells were co-transfected with myc-tagged ALK3 and HA-tagged UBE3A or GFP as a control. Lysates from transfected cells were immunoprecipitated with anti-HA and anti-IgG as a control, followed by western blotting to detect the presence of HA- and myc-tagged proteins. (B) Increased levels of the endogenous pSmad protein and ALK3 were observed in HEK293 cells expressing reduced levels of *UBE3A* by two independent siRNAs. (C) A decreased level of the endogenous ALK3 protein was observed in UBE3A-overexpressing cells; this effect was abolished by treatment with the proteasome inhibitor MG132. (D) Statistical analysis of pMAD and ALK3 protein levels in HEK293 cells expressing reduced levels of *UBE3A* by two independent siRNAs (*n* = 4, one-way ANOVA test, mean ± SEM, **P < 0.01). (E) HA-tagged GFP, wild-type or C833A UBE3A was co-expressed with myc-ALK3 and HA-Ub in HEK293 cells as indicated. Lysates from transfected cells were immunoprecipitated with anti-myc and the levels of ubiquitination were analyzed with an anti-ubiquitin antibody.

Given that Ube3a is evolutionarily conserved from *Drosophila* to vertebrates, we hypothesized that human UBE3A would interact with and negatively regulate ALK3, a mammalian homolog of Tkv [43]. To test this possibility, we examined the co-immunoprecipitation of ALK3 with UBE3A in lysates of human HEK293 cells. An anti-HA antibody, but not a control anti-IgG, co-precipitated myc-ALK3 with HA-UBE3A (Fig 9A). UBE3A knockdown in HEK293 cells using two different small interfering RNAs (siRNAs) produced a significantly increased level of endogenous ALK3 as well as a near two-fold increase in the level of pSmad; the mammalian counterpart of *Drosophila* pMad (Fig 9B and 9D). Conversely, overexpression of UBE3A in HEK293 cells led to a significant decrease in ALK3 protein level (38.1% of the control; *n* = 3, *P* < 0.001), which was blocked by the proteasome inhibitor MG132 (Fig 9C), implying that human UBE3A promotes the proteasomal degradation of ALK3. Overexpression of wild type but not the ligase dead C833A mutant UBE3A significantly increased ubiquitination of myc-ALK3, while GFP control did not (Fig 9E). Despite the fact that the corresponding K227 in ALK3 is not critical for ubiquitination in HEK293 cells (S10 Fig), consistent with the fact that some proteins have little specificity in ubiquitination sites [44], our results demonstrate that Ube3a-mediated suppression of BMP signaling is conserved in mammalian cells.

## Discussion

### Ube3a regulates synapse growth and endocytosis by inhibiting BMP signaling

The synapse is a critical substrate for cognitive function, so disorders associated with intellectual and cognitive deficits, such as AS and autism, likely involve altered pathways that disrupt synaptic development and plasticity. The present study shows for the first time that *ube3a* regulates synapse formation and synaptic endocytosis at the *Drosophila* NMJ. A previous study reported normal NMJ growth in *ube3a*^*15B*^ mutants [35]. However, we showed here that the genetic background in homozygous *ube3a*^*15B*^ mutants may suppress expression of satellite phenotype, as trans-allelic *ube3a*^*15B*^/*ube3a*^*35*^ and hemizygous *ube3a*^*15B*^/*Def* mutants showed excess satellite boutons (S3 Fig). We noted that the satellite phenotype in *ube3a* mutants is mild compared to the classical endocytic *endophilin* and *dap160*/*intersectin* mutants [32, 45, 46]. Also, the fewer and larger SVs at active zones accompanied by an increase in amplitudes of mEJPs reported in endocytic mutants such as *tweek* and *dap160*/*intersectin* mutants [45–47], were not observed in *ube3a* mutants. However, the significantly higher number of satellite boutons, reduced dye uptake, and defective recovery of EJP amplitudes under high-frequency stimulation, implicate a regulatory role for *ube3a* in endocytosis at NMJs. It is worth noting that a similar endocytic function has been reported for mammalian *Ube3a* at inhibitory synapses in the visual cortex [14].

There appears to be a reciprocal regulation between BMP signaling and endocytosis at the *Drosophila* NMJ. Endocytic mutants show upregulated BMP signaling as demonstrated by increased pMad staining at NMJs [40]. Trio, a Rac GTPase-specific GEF, is positively regulated by pMad at the transcriptional level in motoneuron nuclei for proper NMJ development [48]. Conversely, upregulation of BMP signaling by mutations in Dad, an inhibitory Mad that negatively regulates BMP signaling, and in S6KL (S6 kinase like) results in apparent endocytic defects [33, 34]. In addition to the mutual regulation between the two processes, the multi-domain actin regulator Nwk functions at the interface of endocytosis and BMP signaling by physically interacting with both Tkv and endocytic proteins dynamin and Dap160/intersectin [40]. We found normal expression of endocytic proteins in *ube3a* mutant NMJs (S11 Fig), and endocytic *endophilin A* mutants have been shown previously to have a normal level of Tkv [34]. Thus, we favor a model in which upregulated BMP signaling leads to overgrown NMJs and endocytic defects in *ube3a* mutants, and the NMJ defects in *ube3a* mutants could be attributable to altered actin-cytoskeletal formation via Trio-Rac-mediated and/or Nwk-mediated pathways.

While *ube3a* mutation leads to an elevated level of BMP signaling, neuronal overexpression of Ube3a by *elav-Gal4* or *OK6-Gal4* does not result in downregulation of BMP signaling. This was demonstrated by overgrown NMJ terminals and a normal level of pMad at both NMJs and motoneuron nuclei (S12 Fig). These results indicate that overexpression of Ube3a alone is not sufficient to downregulate BMP signaling in the neuromusculature. How Ube3a overexpression leads to overgrown NMJs remains to be determined. Similar overgrown NMJ phenotypes caused by Ube3a mutation and overexpression are consistent with previous reports of defective locomotion [24] and circadian rhythms [25] upon bidirectional changes in Ube3a expression.

### Ube3a ubiquitinates and promotes proteasomal degradation of the BMP receptor Tkv

Using a series of biochemical assays, we showed that Ube3a physically interacts with Tkv and facilitates its proteasome-mediated degradation (Figs 6 and 7). This function of Ube3a was conserved in mammalian cell cultures (Fig 9). We identified K227 but not K218 in the cytoplasmic region of Tkv as being critical for its ubiquitination (Fig 8). We demonstrated that Ube3a normally functions specifically to downregulate Tkv expression by proteasomal degradation, resulting in reduced presynaptic BMP signaling. In the absence of Ube3a, BMP signaling is upregulated, resulting in overgrown NMJs and endocytic defects.

The impact of Ube3a on Tkv is limited to the NMJ, as altered expression of Ube3a does not lead to corresponding changes in Tkv expression in wing discs, where the role of Tkv is well known (S2 Fig). Thus, the negative regulation of Tkv by Ube3a occurs in a tissue specific manner.

In which neuronal compartment is Tkv ubiquitinated? Given that Ube3a (S1 Fig), RING finger E3 ligase Highwire [49], E3 ligase anaphase promoting complex/cyclosome (APC/C) [50], and 20s proteasome subunit [51] are all enriched at NMJ synapses, we favor the possibility that Ube3a ubiquitinates Tkv at presynaptic terminals.

We recently identified that an evolutionarily conserved, previously uncharacterized S6KL similarly regulates NMJ growth and endocytosis by promoting proteasomal degradation of Tkv [34]. As Ube3a biochemically and genetically interacts with S6KL (S13 Fig), it remains possible that Ube3a and S6KL work synergistically in inhibiting NMJ growth by targeting Tkv for degradation

In addition to Ube3a, another HECT-domain-containing E3 ligase Smurf also inhibits BMP signaling by targeting two independent signaling components, BMP receptors and downstream effector Smad in distinct developmental processes [43, 52, 53]. However, Smurf does not regulate NMJ growth [34]. As the protein level of Mad was mildly but significantly increased in *ube3a* mutant brains (Fig 5C and 5D), it would be of interest to determine if Ube3a also targets Mad for proteasome-mediated degradation in NMJ development.

In addition to its role as an ubiquitin E3 ligase, UBE3A has been reported to affect nuclear hormone receptor-mediated transcription by E3 ligase-independent mechanisms [21, 54]. For instance, UBE3A was shown to inhibit the expression of the cyclin-dependent kinase inhibitor p27 at both transcriptional and post-translational levels [55]. Moreover, UBE3A inhibits estradiol-mediated transcription of the target gene *Arc* [21]. As with mammalian UBE3A, *Drosophila* Ube3a also acts as a transcriptional regulator; it activates transcription of *Punch*, which encodes GTP cyclohydrolase 1, a component of the monoamine biosynthesis pathway [27]. In support of a transcriptional role, Ube3a orthologs of different species are localized in the nucleus [16, 23, 27]. However, the present study reveals that Ube3a functions as an ubiquitin ligase for Tkv but not as a transcriptional regulator since the level of *tkv* mRNA in *ube3a* brains was similar to wild type (Fig 5G).

### Involvement of BMP signaling upregulation in the pathophysiology of AS

Human UBE3A negatively regulated the protein levels of ALK3, and consequently the downstream effector pSmad, in HEK293 cells (Fig 9). Therefore, it is tempting to speculate that elevated BMP signaling may contribute to a subset of the abnormalities observed in patients with AS. BMP receptors are expressed at specific sites, such as the hippocampus and cerebellum, where Ube3a is also expressed [10, 56]. It remains to be determined if BMP signaling is upregulated in these brain regions that correlate with the clinical features of seizures, learning deficit and ataxia in patients with AS.

BMP signaling regulates synapse development specifically at the calyx of Held in the auditory system in a way similar to that at *Drosophila* NMJ, but shows no effect on synapse development in the lateral superior olive of the auditory nucleus [31]. Given that AS mice show an enhanced seizure-like response to audiogenic challenge [22], it would be improtant to determine if the development and function of the calyx of Held synapses are normal in *Ube3a* mutant mice. At the microscopic level, AS model mice exhibit normal dendritic elaboration but fewer and shorter dendritic spines in cerebellar Purkinje cells and pyramidal neurons in the hippocampus and cortex [16]. It is not known if BMP signaling plays a role in the formation of dendritic spines, although other targets of Ube3a such as Ephexin5 may regulate spine morphogenesis in hippocampal neurons [20].

In summary, we demonstrated that *Drosophila ube3a* plays an important role in regulating synapse formation and endocytosis by inhibiting BMP signaling via proteasome-mediated degradation of Tkv. The negative regulation of BMP signaling by Ube3a is conserved in mammals. These findings indicate that increased BMP signaling resulting from loss of UBE3A during development may contribute, at least in part, to the etiology of AS.

## Materials and Methods

### *Drosophila* stocks

All *Drosophila* stocks were fed standard cornmeal food and maintained at 25°C. *w*^*1118*^ was used as a wild-type control unless otherwise indicated. We generated intragenic *ube3a* deletions (*ube3a*^*8*^ and *ube3a*^*35*^) through P-element-mediated excisions of *EP3214*. Transgenic lines expressing genomic *ube3a*^*+*^ and *ube3a*^*C941A*^ (containing a missense mutation in the codon for the critical catalytic cysteine residue) were from J. Fischer. *UAS-Tkv-GFP* was from T. B. Kornberg. *UAS-DTS5* was a gift from J. Belote via K. Broadie [51]. The EMS-induced nonsense mutations *ube3a*^*549*^ and *ube3a*^*689*^ were obtained from a TILLING (targeting-induced local lesions in genomes) service (http://tilling.fhcrc.org). For tissue-specific rescue experiments, the pan-neural *elav-Gal4* (from Bloomington) and muscle-specific *C57-Gal4* (from V. Budnik) lines were used. RNAi lines P{GD450}v45876 and *Thu3266* were obtained from the Vienna *Drosophila* RNAi Center (VDRC) and Tsinghua University (Beijing, China), respectively. A yellow fluorescent protein (YFP) genetrap line labeling the endogenous Tkv with YFP was obtained from the *Drosophila* Genome Research Center (stock number 115298; Kyoto, Japan). *EP(3)3214*, *Df(3L)ED4470*, *tkv*^*8*^, *tkv*^*K16713*^, *mad*^*12*^, and *wit*^*B11*^ were obtained from the Bloomington Stock Center.

For overexpression studies, UAS-ube3a was obtained by amplifying the full-length cDNA of *ube3a* from a home-made cDNA pool derived from whole adults, and cloned into the vector pUAST. An *UAS-ube3a* insertion in the X chromosome was used in this study.

### Generation of monoclonal antibodies against Ube3a

To generate specific antibodies against Ube3a, a fusion protein containing amino acids 568–973 of *Drosophila* Ube3a with an N-terminal 6×His tag was produced in *E*. *coli*. Mouse monoclonal antibodies produced by clone 8F7 were shown to be specific for Ube3a by western blotting and immunostaining.

### Immunohistochemical analysis

Immunohistochemical analysis of larval NMJ synapses was performed as previously described [57]. The following antibodies were used: rabbit anti-Tkv (1:200; from M. B. O’Connor, University of Minnesota, Minneapolis, Minnesota), anti-CSP (1:1,000, 6D6 from Developmental Studies Hybridoma Bank (DSHB)), rabbit anti-GFP (1:100, #632460 from Clontech), rabbit anti-pMad (1:500; from P. ten Dijke, Leiden University, Leiden, Netherlands), anti-HRP conjugated with FITC or Texas red (1:50, Jackson ImmunoResearch Laboratories), and anti-Ube3a (1:30, 8F7 as described above). All primary antibodies were visualized using fluorophore-conjugated secondary antibodies, including Alexa Fluor 488 or 568-conjugated goat anti-mouse or anti-rabbit IgG (1:1,000, Molecular Probes). To quantify fluorescence intensities from immunostaining, images of the entire muscle 4 NMJ elaboration or motoneuron nuclei were taken at identical settings for all genotypes without overexposure. The intensity of pMad and Tkv-GFP staining in Fig 5 was calculated as gray values normalized to the pMad- and HRP-positive areas, respectively. The gray values were automatically calculated using ImageJ software (NIH; Bethesda, USA) and presented as arbitrary units (a.u.). All images were collected using a Leica SP5 confocal microscope and processed with Adobe Photoshop.

For statistical analysis of NMJ morphology, the number of total boutons and satellite boutons of muscle 4 NMJ in abdominal segments A2 and A3 was quantified as previously described [57].

### Electrophysiological and FM1-43 uptake assays

Electrophysiological recordings were performed at 20°C essentially following a conventional procedure with minor modifications [33, 34]. For the basal transmission assay, wandering third-instar larvae were dissected in Ca^2+^-free HL3.1 saline and recorded in HL3.1 saline containing 0.5 mM CaCl_2_. Intracellular microelectrodes with a resistance greater than 5 MΩ filled with 3 M KCl were used for the assay. Excitatory junction potential (EJPs) and miniature EJPs (mEJPs) were recorded from muscle 6 in the abdominal segments A2 or A3 for 120 seconds. EJPs were elicited by low frequency (0.3 Hz) stimulation. We analyzed recordings from muscles cells with physiological resting potentials ≤ -60 mV and input resistances > 5 MΩ. For examining synaptic transmission under tetanic stimulation, synapses were stimulated at 10 Hz for 10 minutes and recorded in a modified HL3.1 saline with 0.5 mM extracellular Ca^2+^. For the FM1-43 uptake assay, we followed a previously published protocol [34, 58].

### Cell culture

S2 cells were cultured in Schneider’s medium (Invitrogen, Carlsbad, USA) and transfected using Cellfectin II reagent (Invitrogen). For the production of double-strand RNA (dsRNA) targeting *ube3a* and *gfp*, DNA fragments were synthesized by PCR using primers containing a T7 promoter sequence (italicized): *ube3a* sense: 5’-*TAA TAC GAC TCA CTA TAG GG*A TTG CCG GAA ACC ACT GAT A-3’ and antisense: 5’-*TAA TAC GAC TCA CTA TAG GG*C TCC GTT CTC AAA TGG TGT G-3’, and *gfp* sense: 5’-*TAA TAC GAC TCA CTA TAG GG*A TGG TGA GCA AGG GCG AGG A-3’ and antisense: 5’-*TAA TAC GAC TCA CTA TAG GG*C TTG TAC AGC TCG. The dsRNAs were transcribed from both strands of the PCR-amplified DNA fragments using a Megascript T7 kit (AM1333; Invitrogen), and purified using an RNeasy kit (Qiagen).

To examine Tkv protein turnover, S2 cells were transfected with dsRNAs against *ube3a* or *gfp* (control) at an average transfection efficiency of about 46%, and treated 48 h later with 40 μg/ml cycloheximide (Sigma) for 0, 1.5, 3, 6, and 9 h. To determine whether the degradation of Tkv was proteasome-dependent, S2 cells were co-transfected with flag-Ube3a or flag-GFP plasmid together with myc-Tkv. After 48 h, cells were treated with 20 μM MG132 (Sigma) or mock treated for 6 h before being harvested for western blotting.

Human HEK293 cells were maintained in Dulbecco’s modified Eagle’s medium supplemented with 10% fetal bovine serum (Invitrogen) at 37°C in a humidified incubator with 5% CO_2_. For efficient RNAi knockdown of human UBE3A, *UBE3A* siRNA1 (5’-GGG UCU ACA CCA GAU UGC UTT-3’) [59], siRNA2 (5’-CAA CUC CUG CUC UGA GAU ATT-3’) [60], and a scrambled siRNA control (5’-UUG CGG GUC UAA UCA CCG ATT-3’) were synthesized and transfected into HEK293 cells using X-tremeGENE siRNA transfection reagent (Roche). *UBE3A* and *ALK3* (also named *BMPR1A*) clones were provided, respectively, by Dr. Peter M Howley and William C. Hahn at Harvard Medical School (Cambridge, USA). UBE3A and ALK3 coding sequences were subcloned to generate pCMV-Tag2B-HA-UBE3A and pCDNA3-myc-ALK3, respectively.

### Immunoprecipitation (IP) and western blotting

IP assays of larval brains and ventral ganglia were carried out according to a previous report [61]. Immunoprecipitation from S2 or HEK293 cell lysates was performed following a previously described protocol [43]. Antibodies used for western blotting were: anti-myc (1:1000; 9E10 from Clontech), anti-flag (1:1,000; M2 from Sigma), anti-K48 (1:1,000; D9D5 from Cell Signaling Technologies), anti-GFP (1:1,000), anti-Ube3a (1:300), anti-HA (1:1,000; 3F10 from Roche), anti-ubiquitin (1:1000; P4D1 from Cell Signaling Technologies), anti-Wit (1:50; DSHB), anti-pSmad (1:1000; Cell Signaling Technologies), and anti-α-tubulin (1:25,000; mAb B-5-1-2 from Sigma).

To analyze protein levels in different genotypes, larval brains and ventral ganglia were homogenized in ice-cold lysis buffer (50 mM Tris-HCl (pH 7.4), 150 mM NaCl, 1% (v/v) NP-40, 0.1% (w/v) SDS, 1% (v/v) proteinase inhibitor). Western blots were probed with primary antibodies anti-Ube3a, anti-pMad (1:500, Cell Signaling Technologies), anti-Wit, anti-GFP, and anti-α-tubulin, followed by HRP-conjugated secondary antibody (1:25,000; Sigma).

### *In vivo* and *in vitro* ubiquitination assay

For *in vivo* ubiquitination assay, S2 cells were transfected with plasmids using Cellfectin II reagent (Invitrogen). The ubiquitination assay was performed according to the protocols described previously [43]. In brief, at 48 hr posttransfection, MG132 was added into the media at the final concentration 50μM. Cells were harvested 4 hr later and lysed with a lysis buffer (50 mM Tris [pH 7.5], 120 mM NaCl, and 0.5% NP40) containing 1% (w/v) SDS to disrupt protein–protein interaction and boiled for 10 min, then diluted 10 times with cell lysis buffer.

To detect the levels of ubiquitinated Tkv in vivo, 200 larval brains of each genotype (*elav-Gal4; tkv-GFP and elav-Gal4; tkv-GFP; ube3a*^*35*^) were dissected and incubated with 50 μM MG132 (Sigma) in Schneider’s medium for 4 h to inhibit proteasome-mediated degradation before proceeding with IP. The brains were then homogenized in lysis buffer and the lysates were immunoprecipitated with anti-GFP. The ubiquitinated Tkv was examined by anti-ubiquitin antibody. For *in vitro* ubiquitination assay, the myc-tagged Tkv C-terminus (myc-TkvC) was synthesized by an *in vitro* translation kit containing rabbit reticulocyte lysates (Promega). Ube3a was fused to GST, then expressed and purified from *E*. *coli*. E1, E2, HA-Ub (all three from Boston Biochem), and the potential substrate myc-TkvC, together with the GST control, the E3 ligase Parkin control, or GST-Ube3a, were incubated at 30°C for 1.5 h in 40 μl ubiquitination reaction buffer (50 mM Tris-HCl (pH 7.5), 1 mM dithiothreitol, 50 mM NaCl, 5 mM MgCl_2_, 2 mM ATP). Reactions were terminated by adding SDS-PAGE sample buffer and analyzed by western blotting.

### Mass spectrometry for identification of ubiquitination sites

Mass spectrometry was performed as described previously [62]. myc-Tkv expressed in S2 cells was immunoprecipitated with an anti-myc antibody. The target protein was excised, digested with trypsin, fractionated by HPLC, and analyzed by a LTQ Orbitrap Elite mass spectrometer (ThermoFisher Scientific, Waltham, MA). To further verify the sites of Tkv targeted by Ube3a, myc-Tkv mutants K218R and K227R were generated by site-directed mutagenesis.

### Quantitative RT-PCR of *tkv* mRNA

Total RNA was extracted from brains and ventral nerve ganglia of 40 third instar larvae following the standard Trizol reagent protocol (Invitrogen). Power SYBR Green PCR Master Mix (Applied Biosystems) was used for quantitative real-time PCR. Actin mRNA level was amplified as an internal control using published primers [33], whereas *tkv* cDNA was amplified with primers 5’-GTG ATA GGG CAG GGC GTA GT-3’ and 5’-AGT GGG TCT CGT TCT GTG GG-3’.

### Statistical analysis

Student’s *t* tests were used for statistical comparisons between two groups. Multiple group means were compared by one-way ANOVA. Asterisks above a column indicate comparisons between a specific genotype and wild type, whereas asterisks above a bracket denote comparisons between two specific genotypes. Data are presented as mean ± standard error of the mean (SEM). *P*-values < 0.05 were considered statistically significant.

## Supporting Information

S1 Fig Ube3a is cytoplasmic and widely expressed in *Drosophila*. (A) Ube3a protein is cytoplasmic and is highly expressed in the ventral ganglion of a third instar larva. The ventral ganglion was double-labeled with a monoclonal antibody 8F7 against Ube3a (green) and propidium iodide (magenta) to visualize nuclei. (B) No specific Ube3a staining was detected in *ube3a*^*35*^ ventral ganglion. (C) Ube3a is expressed in muscles and axons with specific enrichment at presynaptic NMJ terminals compared with background expression of Ube3a in mutants. Larval preparations were double-stained with anti-Ube3a (green) and anti-HRP (magenta). Ube3a is cytoplasmic and expressed in the adult ovary (D) and adult testis (E).
(TIF)

S2 Fig Ube3a antibody is specific for immunostaining and Ube3a expresses in motoneurons. (A, B) The specificity of anti-Ube3a was demonstrated by staining wing disc expressing a reduced level (*en-Gal4/+;* RNAi /+; A) or an upregulated level (*UAS-ube3a/+; en-Gal4/+*; B) of Ube3a in the posterior compartment by *en-Gal4*. Tkv protein level remains unchanged upon altered expressions of Ube3a. Scale bar = 40 μm. (C) The ventral ganglion of a *OK6-Gal4/UAS-CD8-GFP* larva was double-labeled with GFP (bright enough without staining) and anti-Ube3a. Ube3a expresses in GFP-positive motoneurons as well as GFP negative interneurons. (D) No specific Ube3a staining was detected in *ube3a*^*35*^ ventral ganglion. Scale bar = 40 μm.
(TIF)

S3 Fig *ube3a* regulates NMJ growth presynaptically. (A) Muscle 4 NMJ synapses were double-stained with anti-HRP (green) and anti-CSP (magenta). *OK6-Gal4*-driven *Thu3266* RNAi against *ube3a* in motoneurons led to more total boutons and satellite boutons compared with the control. Scale bar = 10 μm. (B) Quantification of total boutons and satellite boutons in control (*OK6-Gal4/+*) and *OK6-Gal4/+*; *RNAi/+* animals. *n* ≥ 16 NMJs; ***P < 0.001 by *t* test; error bars represent SEM. (C) The NMJ growth appeared normal in *ube3a*^*15B*^ homozygotes, but there were more total boutons and more satellite boutons in *ube3a*^*15B*^*/ube3a*^*35*^ and *ube3a*^*15B*^*/Def* mutants compared with *ube3a*^*15B*^. (D) Quantification of the total boutons and satellite boutons in the three genotypes. *n* ≥ 16 NMJs; ***P < 0.001 by one-way ANOVA test; error bars represent SEM.
(TIF)

S4 Fig More cisternae in the presynaptic boutons of *ube3a* mutants. (A, B) Electron micrographs of synaptic boutons from wild type (A) and *ube3a*^*35*^ mutants (B). Compared with wild type, *ube3a*^*35*^ mutants exhibited significantly more cisternae with diameters >60 nm (arrowheads in B). Arrows in A and B indicate active zone. Scale bar, 500 nm. (C, D) High magnification view of representative active zones from wild type (C) and *ube3a*^*35*^ mutants (D). Dashed line defines a 200 nm radius around the active zone for quantitative analysis of SVs. Scale bar, 100 nm. (E) High magnification view of a presynaptic bouton from *ube3a*^*35*^ mutants. Arrowheads indicate cisternae. Scale bar, 100 nm. (F, G) Quantification of the number of SVs within a 200 nm radius of the active zone (F) and the mean number of cisternae per cross-sectioned presynaptic bouton (G). *n* ≥ 38 boutons from more than 4 larvae analyzed, *t* test, mean ± s.e.m., ***p < 0.001.
(TIF)

S5 Fig *ube3a* mutants show normal amplitudes of EJP and normal frequency and amplitudes of mEJP. (A, B) Representative traces of EJP and mEJP of wild type (A) and *ube3a*^*35*^ mutant (B) NMJs. Scale bars for EJP and mEJP are annotated.
(TIF)

S6 Fig The defects of increased pMad level, decreased dye loading and enhanced rundown in *ube3a* mutants are fully rescued by reducing the dose of *tkv* by half. (A, B) Increased levels of pMad at NMJ synapses (A) and motoneuron nuclei (B) of *ube3a*^*35*^ mutants were rescued by a heterozygous mutation of *tkv*^*8*^. Scale bars, 5 μm and 10 μm in A and B, respectively. (C) Quantification of the fluorescence intensities of pMad at NMJ and nuclei of different genotypes including wild type, *ube3a*^*35*^, and *tkv*^*8*^*/+*; *ube3a*^*35*^. ***p < 0.001 by one-way ANOVA; error bars indicate SEM. (D) NMJ4 synapses in abdominal segment A3 loaded with FM1-43 in wild type, *ube3a*^*35*^, and *tkv*^*8*^/*+*; *ube3a*^*35*^. Scale bar, 5 μm. (E) Quantification of FM1-43 intensities in NMJ boutons following high K^+^-stimulated endocytosis. *n* ≥ 14 NMJs; ***p < 0.001 by one-way ANOVA; error bars indicate SEM. (F) Percentage of average EJP amplitudes under tetanic stimulation for 10 min in wild type, *ube3a*^*35*^, and *tkv*^*8*^*/+*; *ube3a*^*35*^. *n* ≥ 10 animals. Representative traces are shown on the right.
(TIF)

S7 Fig Tkv-YFP expresses in motoneurons of a ventral ganglion. (A) The ventral ganglion of a wild-type larva was double-labeled with anti-GFP and anti-HRP. No GFP signals were detected in the ventral ganglion. (B) Tkv-YFP under the control of the endogenous promoter expresses in the soma of various neurons including motoneurons along the midline of a ventral ganglion. Scale bar = 40 μm. (C, D) Tkv-YFP is functional as the genetrap line shows NMJ growth (D) comparable to wild-type control (C).
(TIF)

S8 Fig Delineation of the regions mediating the interaction between Ube3a and Tkv. (A) Schematic representation of full-length and various truncated Ube3a used for co-IP assays. (B) The N-terminal regions of Ube3a mediate the interaction with myc-Tkv. S2 cell lysates were transfected with different combinations of constructs and immunoprecipitated with anti-flag antibody, followed by western blotting using anti-flag and anti-myc. IgG heavy chain is indicated by an asterisk. α-tubulin was used as a loading control. (C) Schematic diagram of full-length Tkv and its various truncations. Different functional domains are indicated. (D) Co-IP assays showed that the C-terminus of Tkv (TkvC), lacking the extracellular and transmembrane (TM) regions, was able to bind Ube3a. More precisely, the dual-specificity serine-threonine/tyrosine protein kinase catalytic (STYKc) domain exhibited strong binding to Ube3a; consistently, deletion of this domain greatly reduced the interaction.
(TIF)

S9 Fig An increased level of pMad at NMJ and motoneuron nuclei upon inhibiting proteasome function by MG132 or expression of DTS5 by *elav-Gal4*. (A) The level of pMad increased at NMJ synapses and motoneuron nuclei of dissected wild-type larvae treated with 50 μM MG132 inhibitor in Schneider’s medium for 4 h. (B) Quantification of fluorescence intensities of pMad at NMJ and nuclei of non-treated and MG1320-treated larvae. *n* ≥ 16; **P < 0.01, ***p < 0.001 by one-way ANOVA; error bars indicate SEM. (C) Expressing DTS5, a dominant temperature-sensitive mutation of the β6 subunit of the 26s proteasome (Speese et al., *Curr Biol*, 2003), by *elav-Gal4* (*elav-Gal4/+*; *DTS5/+*) also led to an increase of pMad at NMJ synapses and the motoneuron nuclei. (D) Statistical analysis of fluorescence intensities of pMad at NMJs and motoneuron nuclei of *elav/+* control and *elav-Gal4-*driven *DTS5* larvae. *n* ≥ 18; **P < 0.01, ***p < 0.001 by one-way ANOVA; error bars represent SEM.
(TIF)

S10 Fig The ubiquitination site K227 of Tkv is not conserved in ALK3. (A) Sequence alignment of Tkv and human ALK3 spanning the ubiquitination site K227. (B) Wild-type and mutant ALK3 showed a similar level of proteins in the presence of UBE3A. Tubulin was probed as a loading control.
(TIF)

S11 Fig Endocytic proteins are expressed normally at ube3a mutant NMJ synapase. Confocal images of NMJ4 from wild type (A, C, and E) and *ube3a*^*35*^ mutants (B, D, and F) labeled with anti-Dynamin (A and B), anti-Eps15 (C and D), and anti-Endophilin A (E and F).
(TIF)

S12 Fig *ube3a* overexpression by *elav-Gal4* leads to overgrown NMJs. (A) Overexpression of Ube3a in neurons by *elav-Gal4* but not in muscles by *C57-Gal4* leads to overgrown NMJ terminals. (B, C) Neuronal overexpression of Ube3a does not drive down BMP signaling as pMad staining appears normal at both NMJs (B) and motoneuron nuclei (C).
(TIF)

S13 Fig Ube3a interacts physically and genetically with S6KL. (A) Different plasmid constructs were singly or co-transfected to S2 cells. Transfected cells were harvested 48 hr later. Cell lysates were used in a two-step immunoprecipitation step by anti-myc and anti-HA sequentially. (B) Confocal images of NMJ4 labeled with anti-HRP. Loss of one copy of *S6KL* or *ube3a* had no effect on the number of satellite boutons, while the number of satellite boutons increased significantly in *S6KL*^*140*^ and *ube3a*^*35*^ trans-heterozygotes and double mutants. (C) Quantification of satellite bouton number in different genotypes. *n* ≥ 16 NMJs analyzed, one-way ANOVA, mean ± s.e.m., ***p < 0.001.
(TIF)

S1 Text Supplementary materials and methods. (DOC)
