# Supplementary figures and images for: Angelman Syndrome Protein Ube3a Regulates Synaptic Growth and Endocytosis by Inhibiting BMP Signaling in Drosophila
Source: PLoS Genet. 2016 May 27;12(5):e1006062. doi: 10.1371/journal.pgen.1006062 (PMC4883773; doi:10.1371/journal.pgen.1006062)

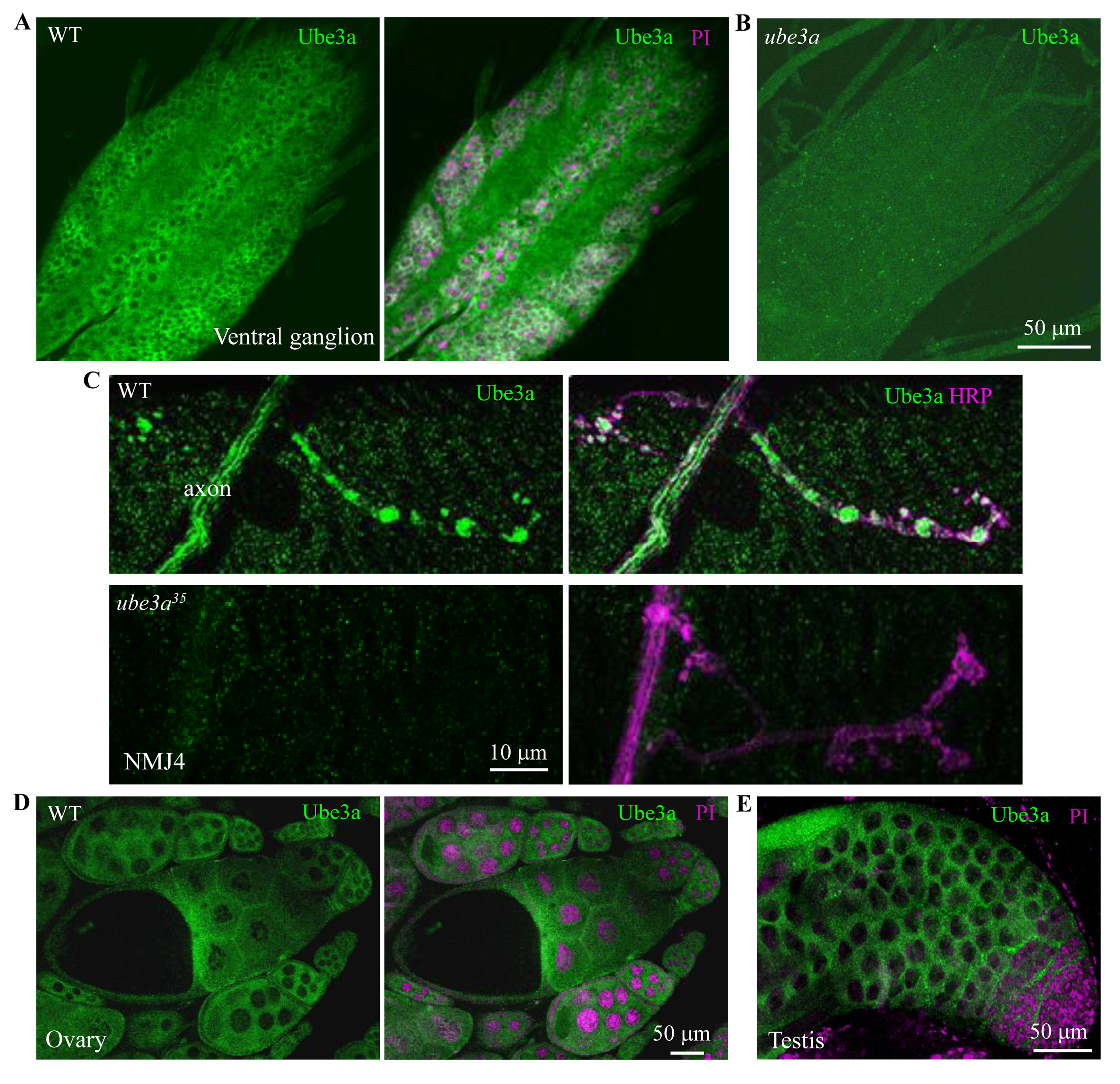

Supplement: S1 Fig — (A) Ube3a protein is cytoplasmic and is highly expressed in the ventral ganglion of a third instar larva. The ventral ganglion was double-labeled with a monoclonal antibody 8F7 against Ube3a (green) and propidium iodide (magenta) to visualize nuclei. (B) No specific Ube3a staining was detected in ube3a35 ventral ganglion. (C) Ube3a is expressed in muscles and axons with specific enrichment at presynaptic NMJ terminals compared with background expression of Ube3a in mutants. Larval preparations were double-stained with anti-Ube3a (green) and anti-HRP (magenta). Ube3a is cytoplasmic and expressed in the adult ovary (D) and adult testis (E). (TIF) [file pgen.1006062.s001.tif]

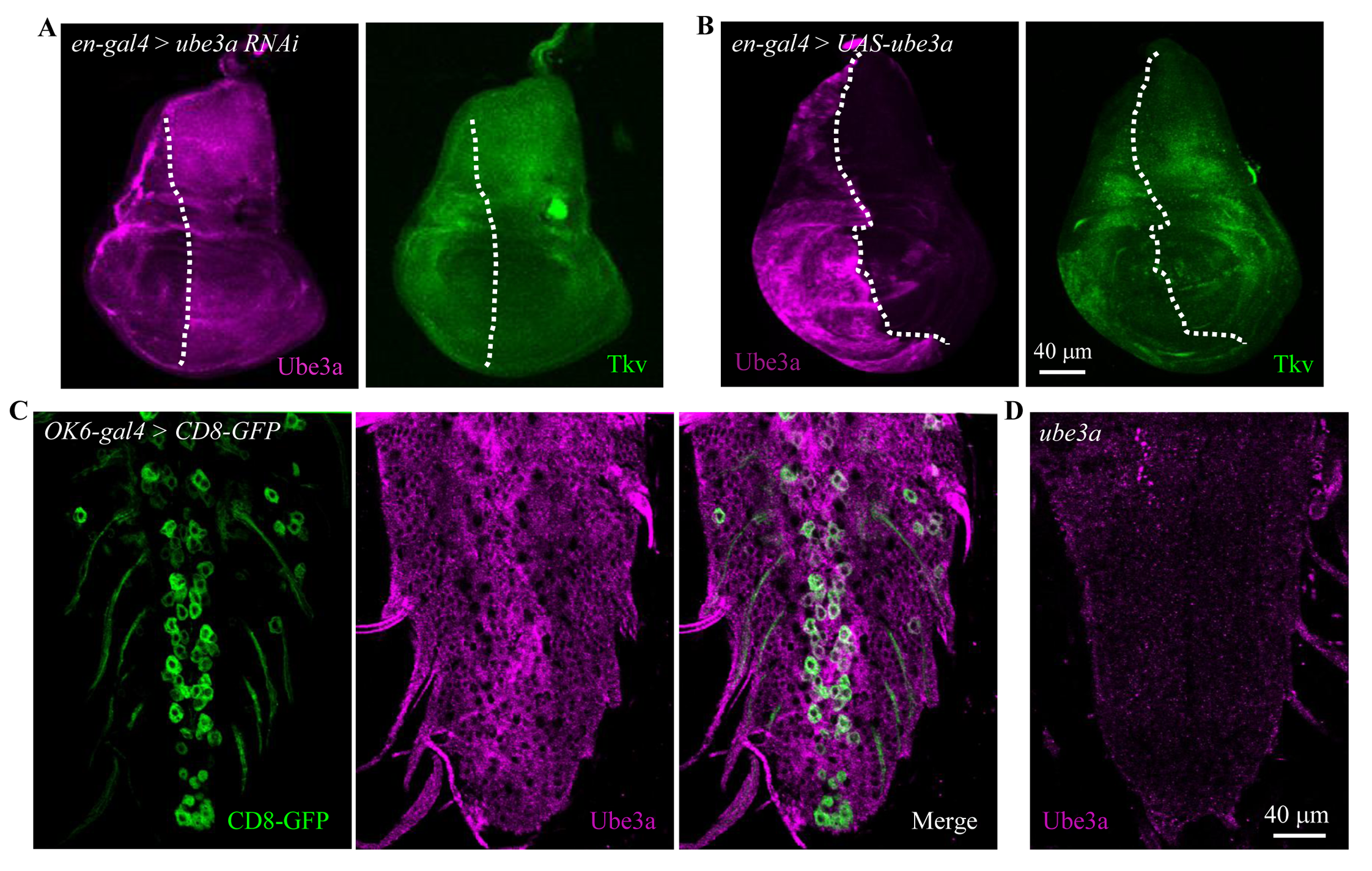

Supplement: S2 Fig — (A, B) The specificity of anti-Ube3a was demonstrated by staining wing disc expressing a reduced level (en-Gal4/+; RNAi /+; A) or an upregulated level (UAS-ube3a/+; en-Gal4/+; B) of Ube3a in the posterior compartment by en-Gal4. Tkv protein level remains unchanged upon altered expressions of Ube3a. Scale bar = 40 μm. (C) The ventral ganglion of a OK6-Gal4/UAS-CD8-GFP larva was double-labeled with GFP (bright enough without staining) and anti-Ube3a. Ube3a expresses in GFP-positive motoneurons as well as GFP negative interneurons. (D) No specific Ube3a staining was detected in ube3a35 ventral ganglion. Scale bar = 40 μm. (TIF) [file pgen.1006062.s002.tif]

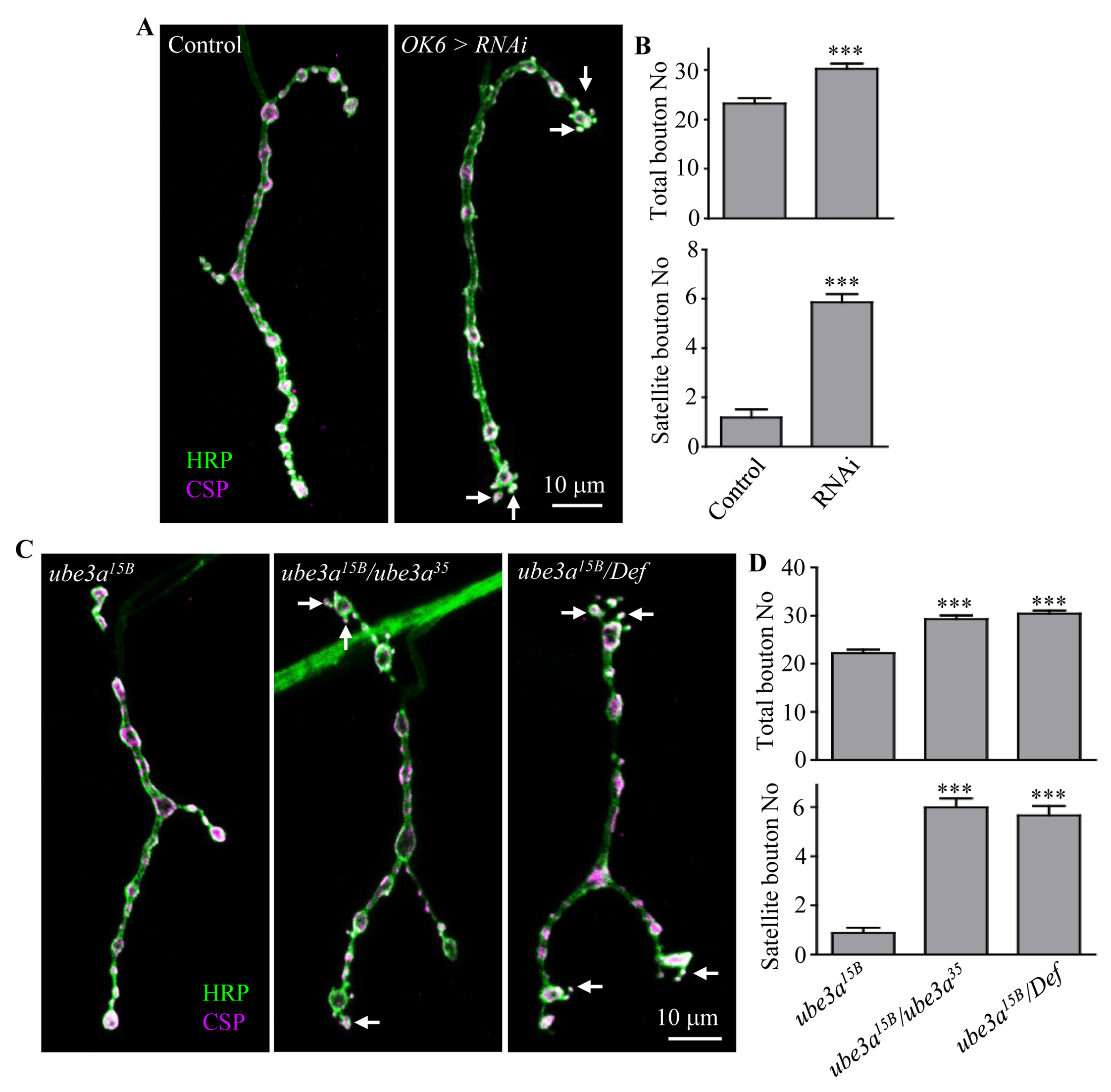

Supplement: S3 Fig — (A) Muscle 4 NMJ synapses were double-stained with anti-HRP (green) and anti-CSP (magenta). OK6-Gal4-driven Thu3266 RNAi against ube3a in motoneurons led to more total boutons and satellite boutons compared with the control. Scale bar = 10 μm. (B) Quantification of total boutons and satellite boutons in control (OK6-Gal4/+) and OK6-Gal4/+; RNAi/+ animals. n ≥ 16 NMJs; ***P < 0.001 by t test; error bars represent SEM. (C) The NMJ growth appeared normal in ube3a15B homozygotes, but there were more total boutons and more satellite boutons in ube3a15B/ube3a35 and ube3a15B/Def mutants compared with ube3a15B. (D) Quantification of the total boutons and satellite boutons in the three genotypes. n ≥ 16 NMJs; ***P < 0.001 by one-way ANOVA test; error bars represent SEM. (TIF) [file pgen.1006062.s003.tif]

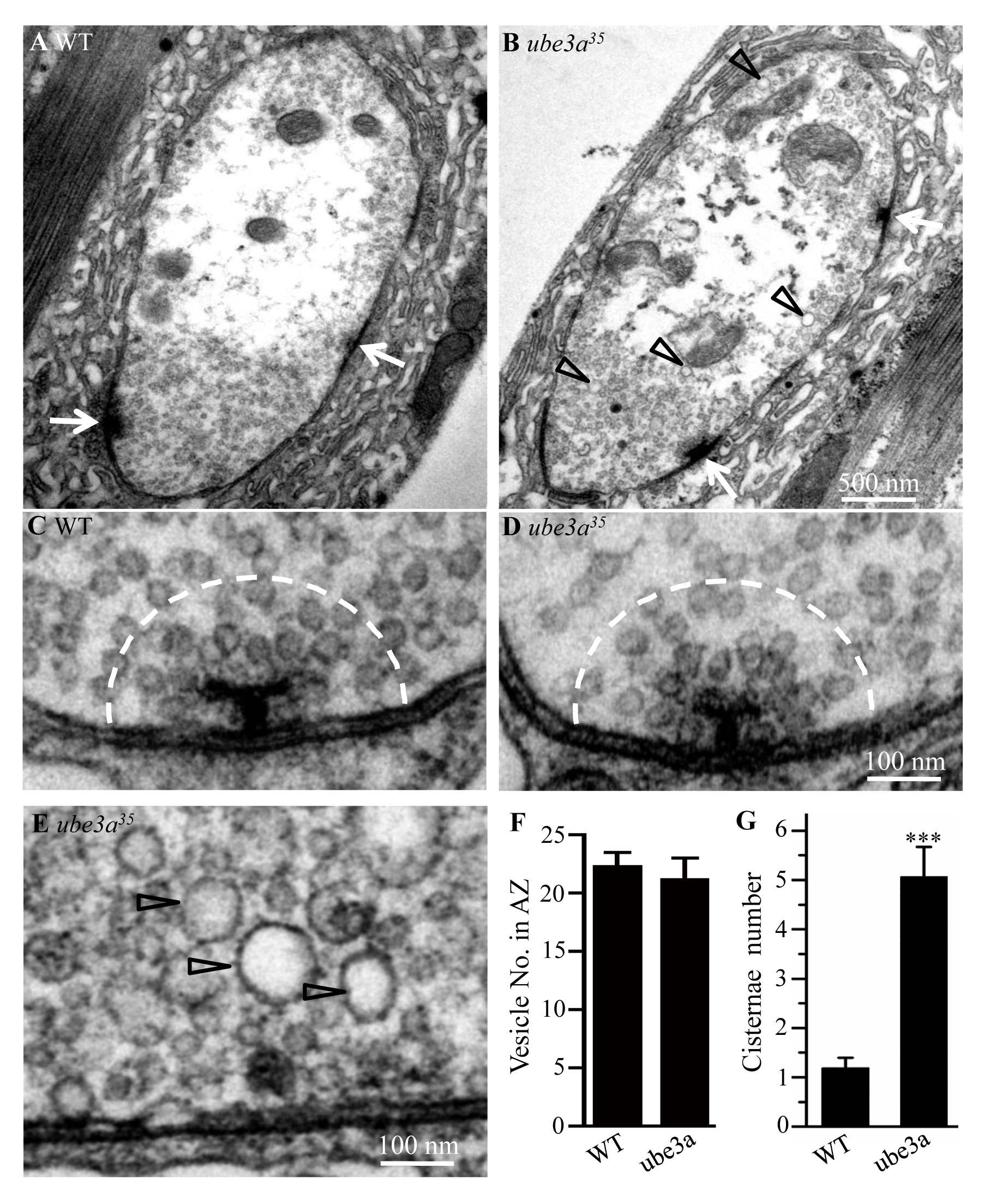

Supplement: S4 Fig — (A, B) Electron micrographs of synaptic boutons from wild type (A) and ube3a35 mutants (B). Compared with wild type, ube3a35 mutants exhibited significantly more cisternae with diameters >60 nm (arrowheads in B). Arrows in A and B indicate active zone. Scale bar, 500 nm. (C, D) High magnification view of representative active zones from wild type (C) and ube3a35 mutants (D). Dashed line defines a 200 nm radius around the active zone for quantitative analysis of SVs. Scale bar, 100 nm. (E) High magnification view of a presynaptic bouton from ube3a35 mutants. Arrowheads indicate cisternae. Scale bar, 100 nm. (F, G) Quantification of the number of SVs within a 200 nm radius of the active zone (F) and the mean number of cisternae per cross-sectioned presynaptic bouton (G). n ≥ 38 boutons from more than 4 larvae analyzed, t test, mean ± s.e.m., ***p < 0.001. (TIF) [file pgen.1006062.s004.tif]

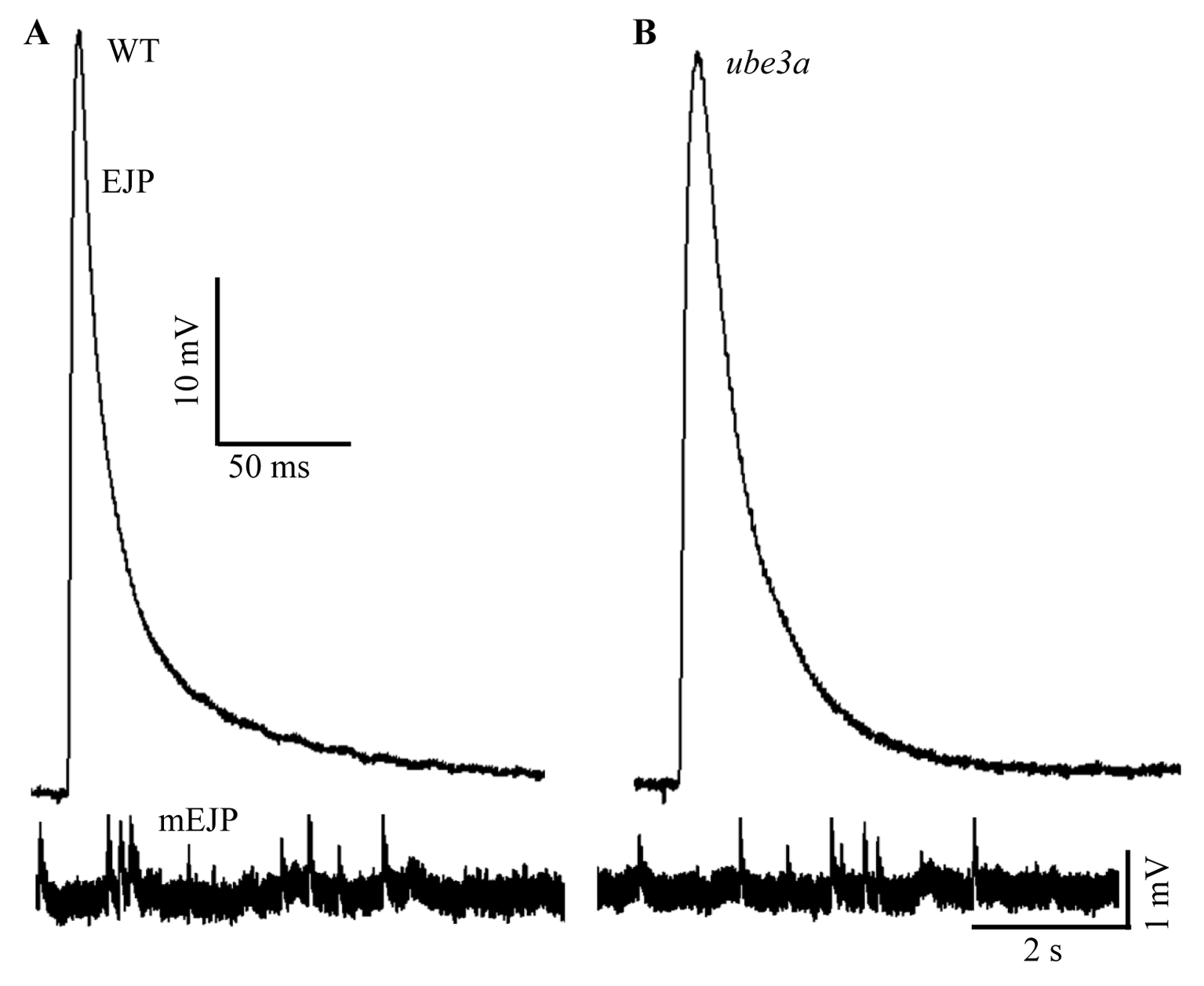

Supplement: S5 Fig — (A, B) Representative traces of EJP and mEJP of wild type (A) and ube3a35 mutant (B) NMJs. Scale bars for EJP and mEJP are annotated. (TIF) [file pgen.1006062.s005.tif]

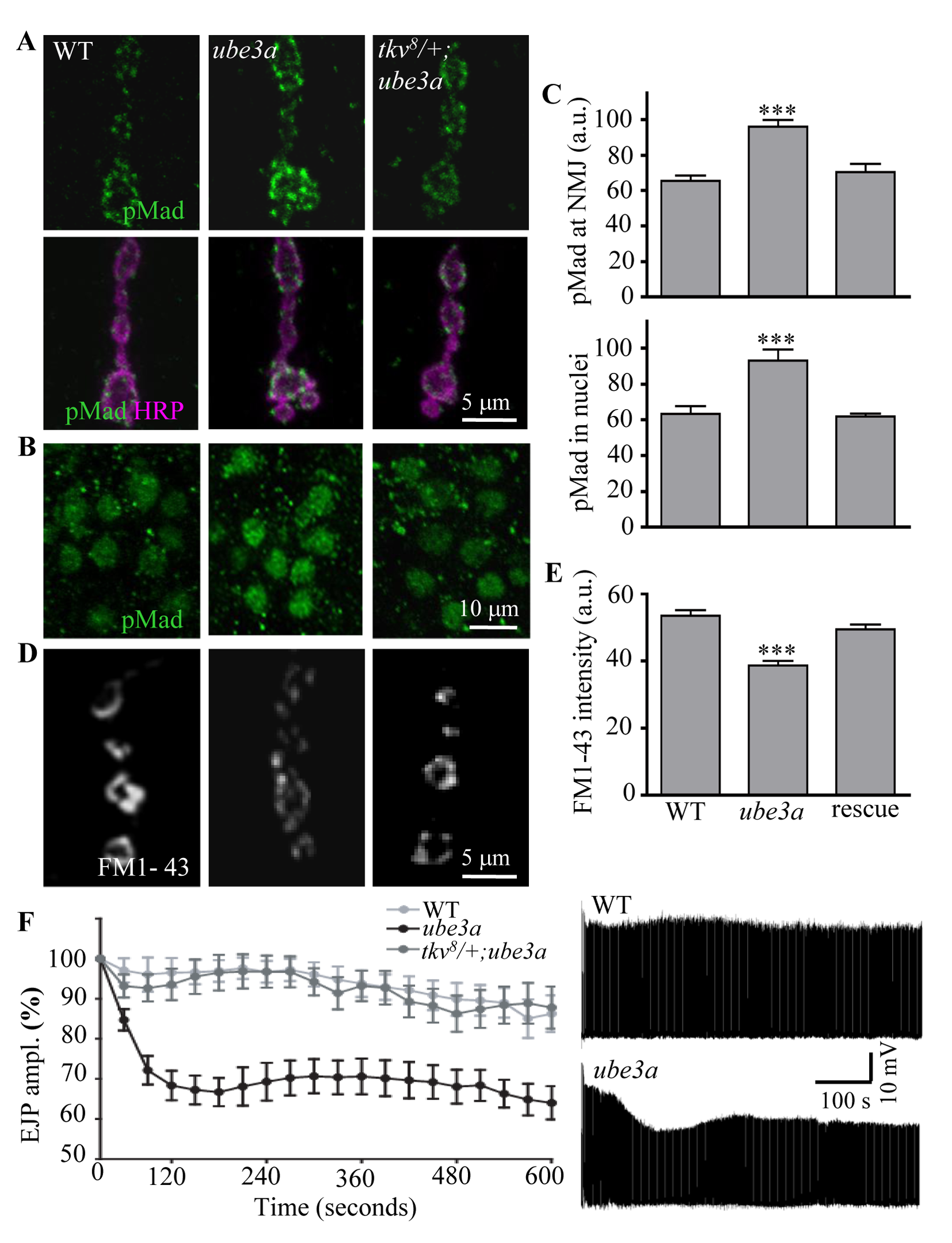

Supplement: S6 Fig — (A, B) Increased levels of pMad at NMJ synapses (A) and motoneuron nuclei (B) of ube3a35 mutants were rescued by a heterozygous mutation of tkv8. Scale bars, 5 μm and 10 μm in A and B, respectively. (C) Quantification of the fluorescence intensities of pMad at NMJ and nuclei of different genotypes including wild type, ube3a35, and tkv8/+; ube3a35. ***p < 0.001 by one-way ANOVA; error bars indicate SEM. (D) NMJ4 synapses in abdominal segment A3 loaded with FM1-43 in wild type, ube3a35, and tkv8/+; ube3a35. Scale bar, 5 μm. (E) Quantification of FM1-43 intensities in NMJ boutons following high K+-stimulated endocytosis. n ≥ 14 NMJs; ***p < 0.001 by one-way ANOVA; error bars indicate SEM. (F) Percentage of average EJP amplitudes under tetanic stimulation for 10 min in wild type, ube3a35, and tkv8/+; ube3a35. n ≥ 10 animals. Representative traces are shown on the right. (TIF) [file pgen.1006062.s006.tif]

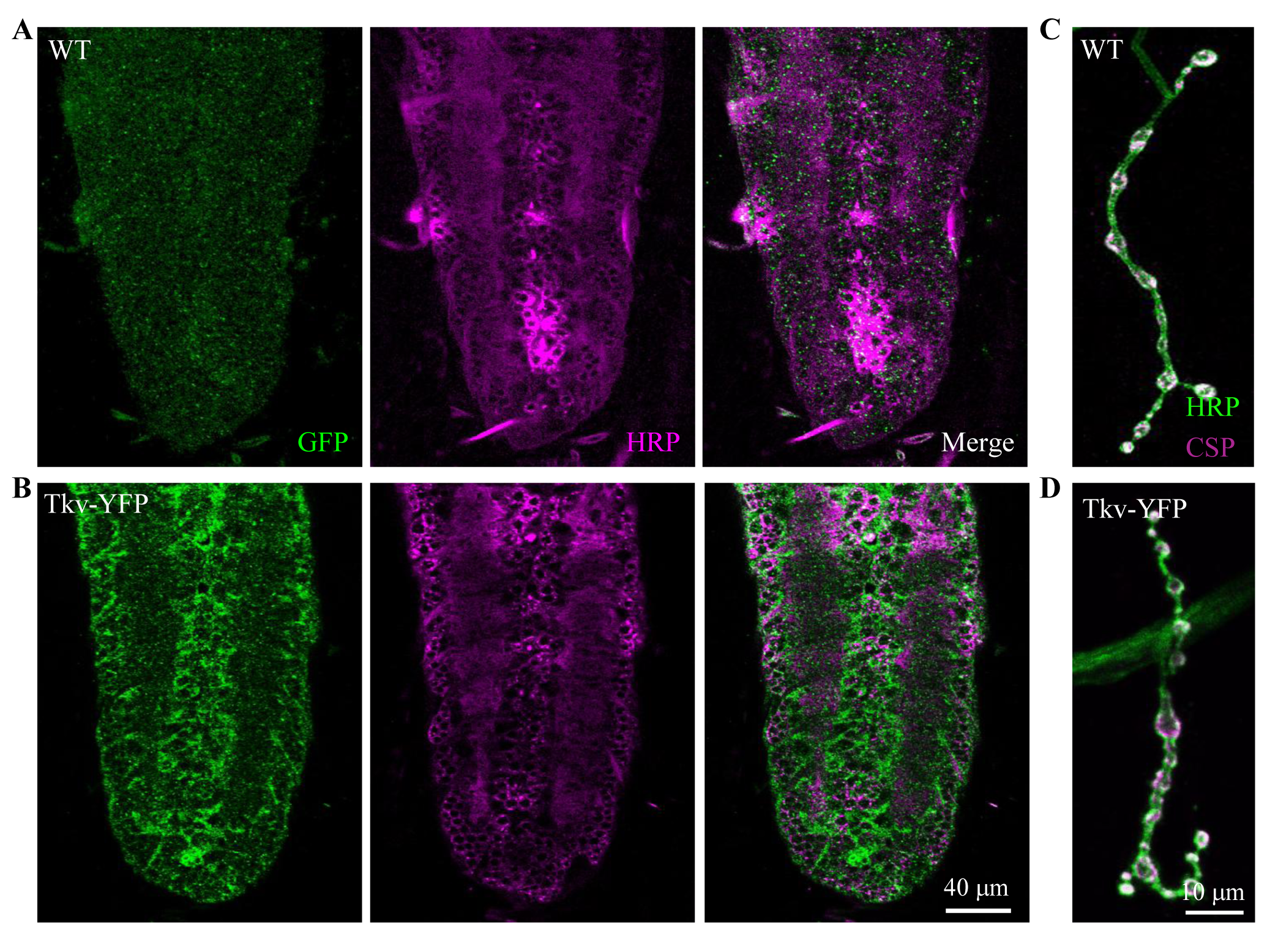

Supplement: S7 Fig — (A) The ventral ganglion of a wild-type larva was double-labeled with anti-GFP and anti-HRP. No GFP signals were detected in the ventral ganglion. (B) Tkv-YFP under the control of the endogenous promoter expresses in the soma of various neurons including motoneurons along the midline of a ventral ganglion. Scale bar = 40 μm. (C, D) Tkv-YFP is functional as the genetrap line shows NMJ growth (D) comparable to wild-type control (C). (TIF) [file pgen.1006062.s007.tif]

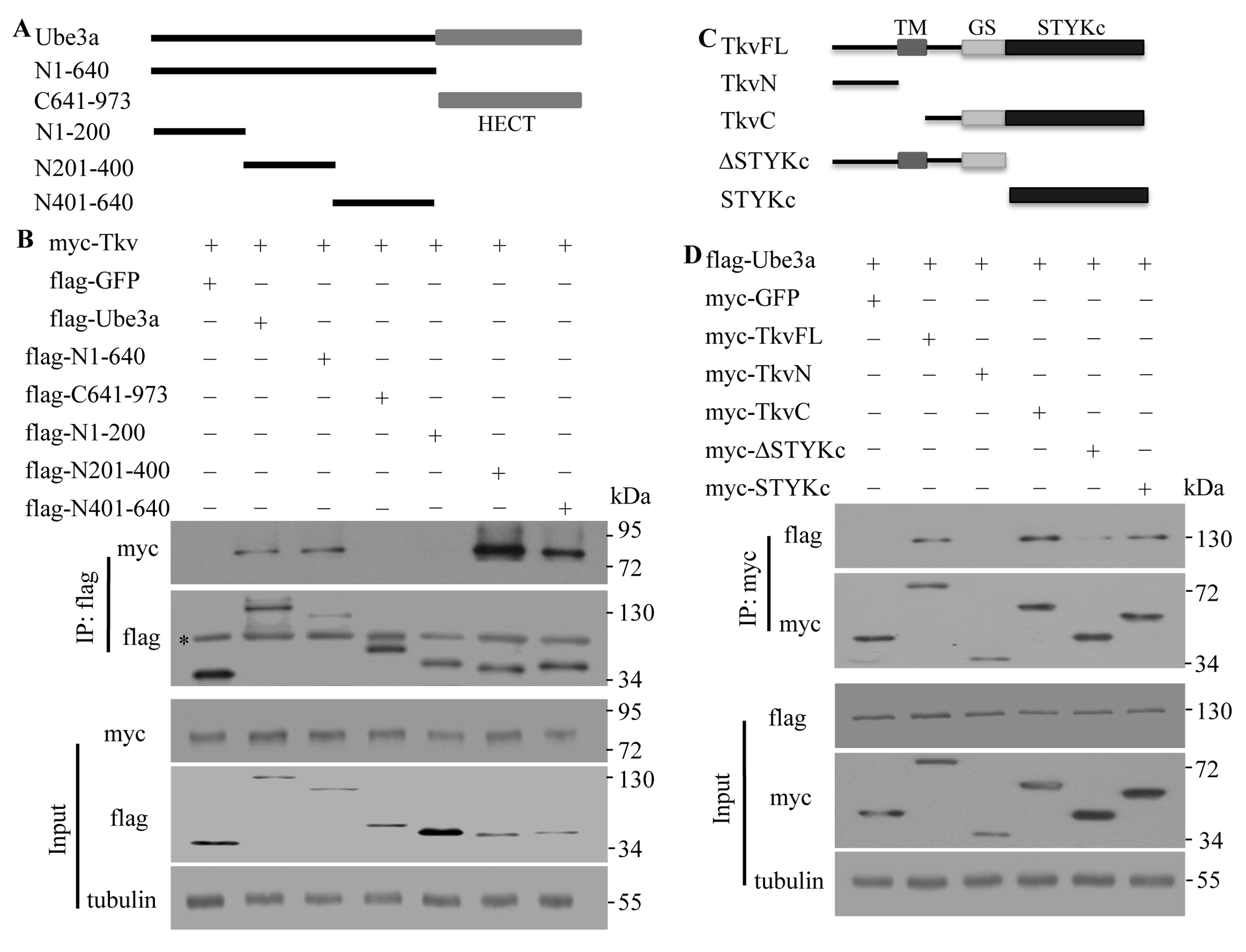

Supplement: S8 Fig — (A) Schematic representation of full-length and various truncated Ube3a used for co-IP assays. (B) The N-terminal regions of Ube3a mediate the interaction with myc-Tkv. S2 cell lysates were transfected with different combinations of constructs and immunoprecipitated with anti-flag antibody, followed by western blotting using anti-flag and anti-myc. IgG heavy chain is indicated by an asterisk. α-tubulin was used as a loading control. (C) Schematic diagram of full-length Tkv and its various truncations. Different functional domains are indicated. (D) Co-IP assays showed that the C-terminus of Tkv (TkvC), lacking the extracellular and transmembrane (TM) regions, was able to bind Ube3a. More precisely, the dual-specificity serine-threonine/tyrosine protein kinase catalytic (STYKc) domain exhibited strong binding to Ube3a; consistently, deletion of this domain greatly reduced the interaction. (TIF) [file pgen.1006062.s008.tif]

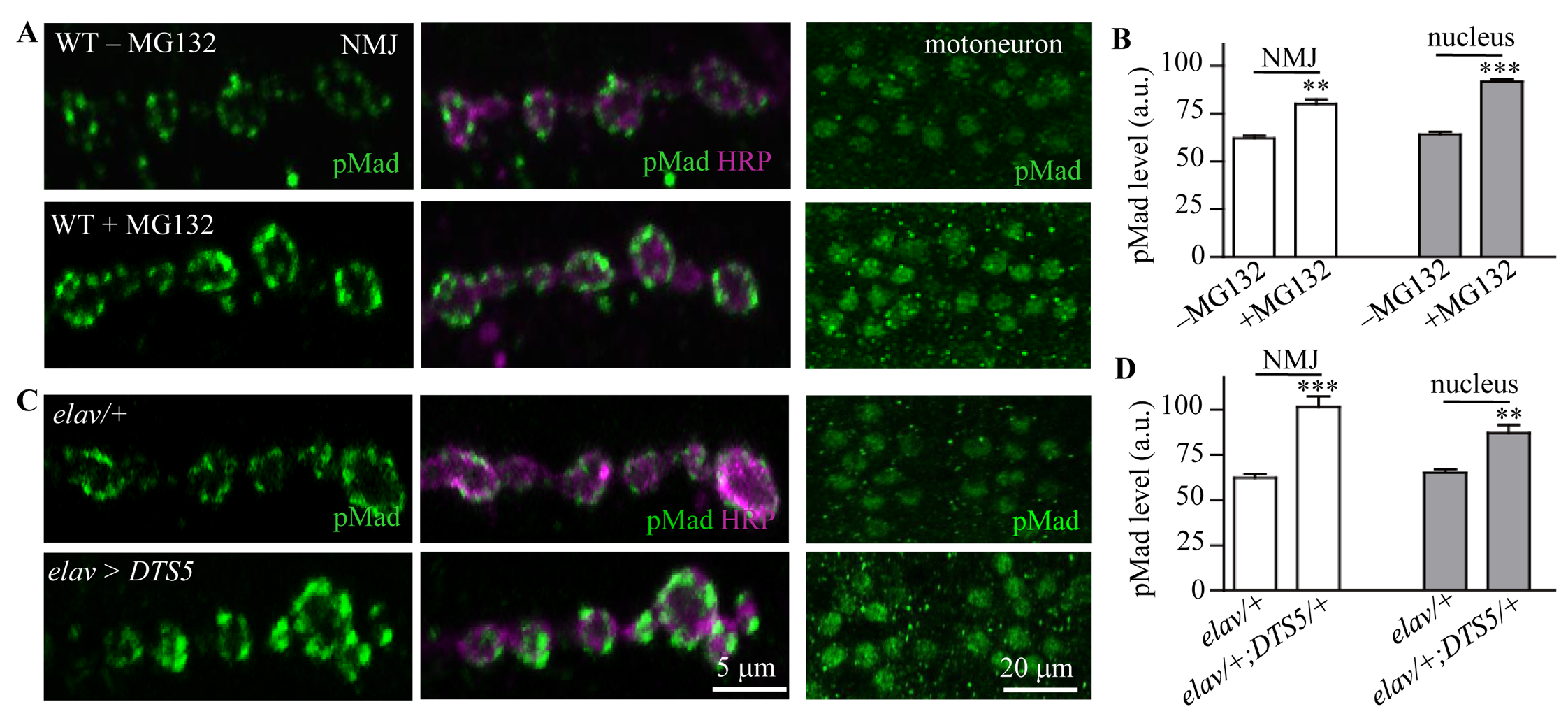

Supplement: S9 Fig — (A) The level of pMad increased at NMJ synapses and motoneuron nuclei of dissected wild-type larvae treated with 50 μM MG132 inhibitor in Schneider’s medium for 4 h. (B) Quantification of fluorescence intensities of pMad at NMJ and nuclei of non-treated and MG1320-treated larvae. n ≥ 16; **P < 0.01, ***p < 0.001 by one-way ANOVA; error bars indicate SEM. (C) Expressing DTS5, a dominant temperature-sensitive mutation of the β6 subunit of the 26s proteasome (Speese et al., Curr Biol, 2003), by elav-Gal4 (elav-Gal4/+; DTS5/+) also led to an increase of pMad at NMJ synapses and the motoneuron nuclei. (D) Statistical analysis of fluorescence intensities of pMad at NMJs and motoneuron nuclei of elav/+ control and elav-Gal4-driven DTS5 larvae. n ≥ 18; **P < 0.01, ***p < 0.001 by one-way ANOVA; error bars represent SEM. (TIF) [file pgen.1006062.s009.tif]

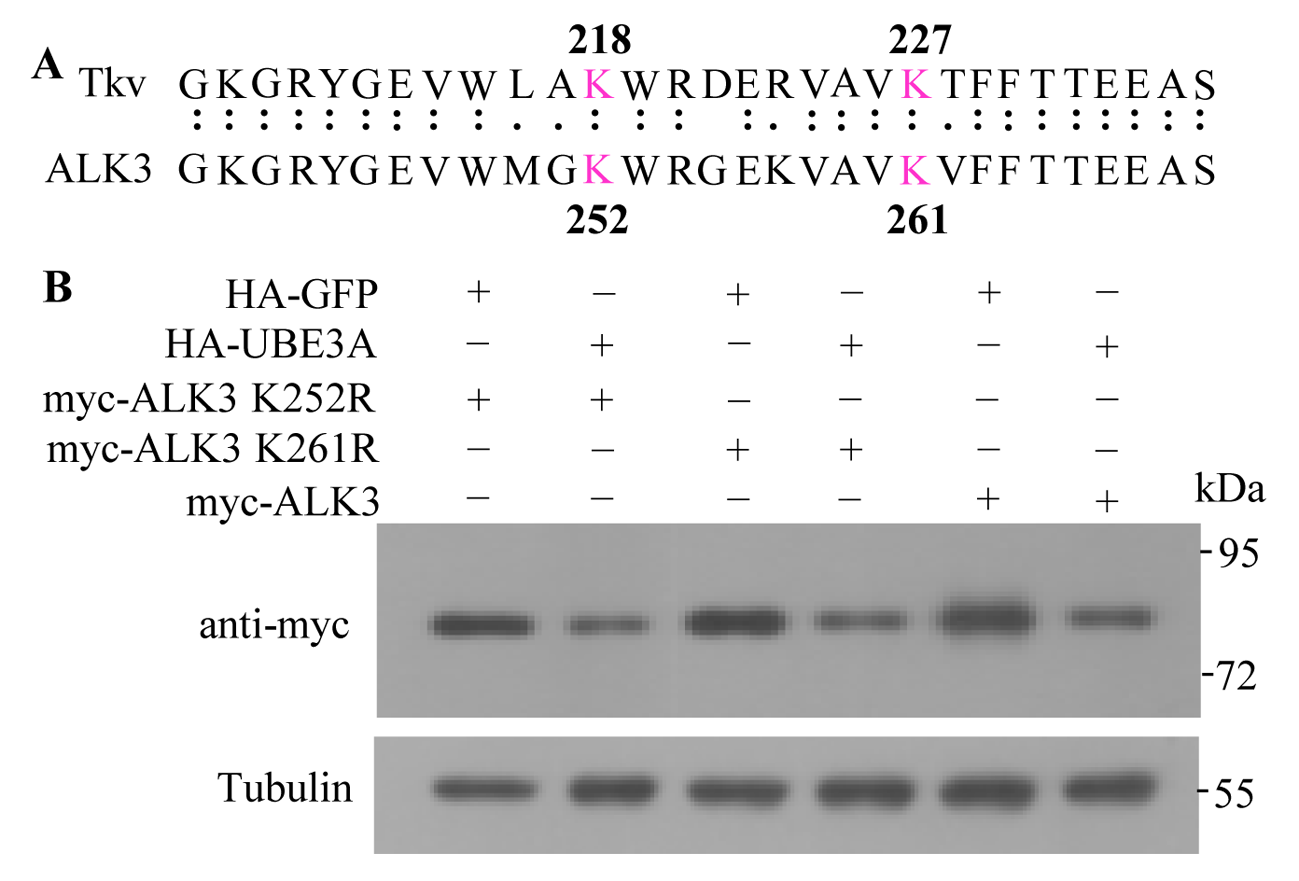

Supplement: S10 Fig — (A) Sequence alignment of Tkv and human ALK3 spanning the ubiquitination site K227. (B) Wild-type and mutant ALK3 showed a similar level of proteins in the presence of UBE3A. Tubulin was probed as a loading control. (TIF) [file pgen.1006062.s010.tif]

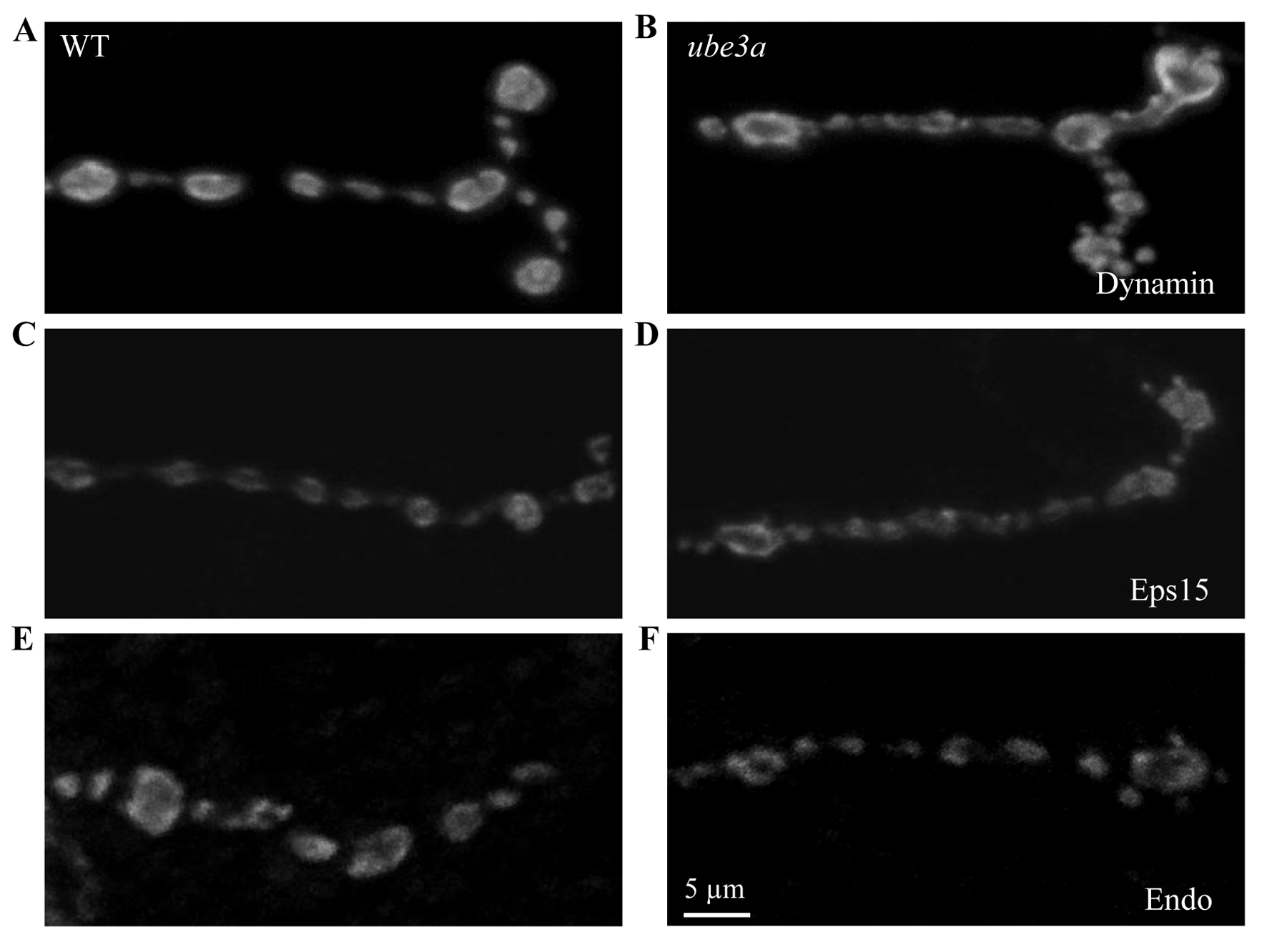

Supplement: S11 Fig — Confocal images of NMJ4 from wild type (A, C, and E) and ube3a35 mutants (B, D, and F) labeled with anti-Dynamin (A and B), anti-Eps15 (C and D), and anti-Endophilin A (E and F). (TIF) [file pgen.1006062.s011.tif]

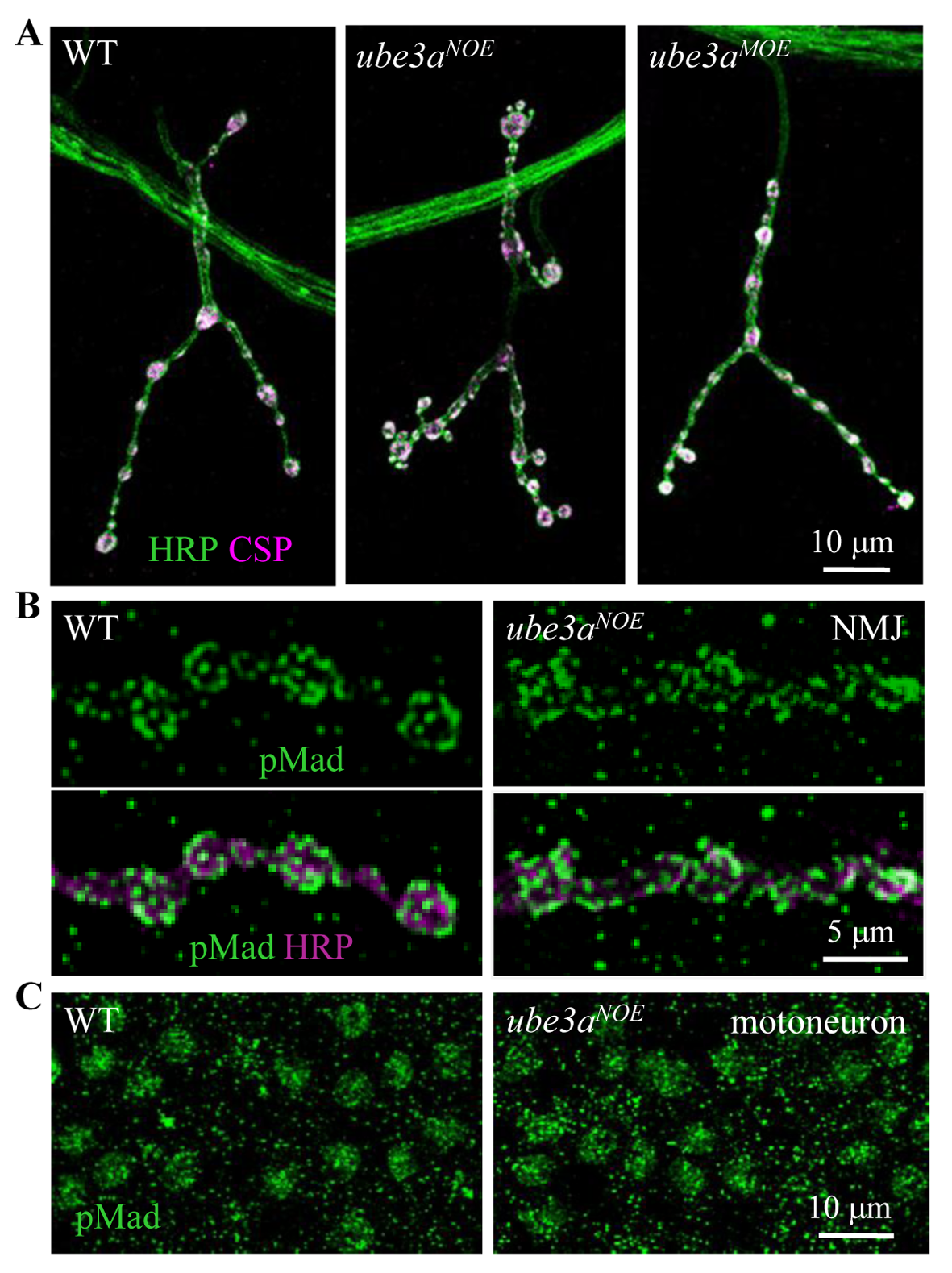

Supplement: S12 Fig — (A) Overexpression of Ube3a in neurons by elav-Gal4 but not in muscles by C57-Gal4 leads to overgrown NMJ terminals. (B, C) Neuronal overexpression of Ube3a does not drive down BMP signaling as pMad staining appears normal at both NMJs (B) and motoneuron nuclei (C). (TIF) [file pgen.1006062.s012.tif]

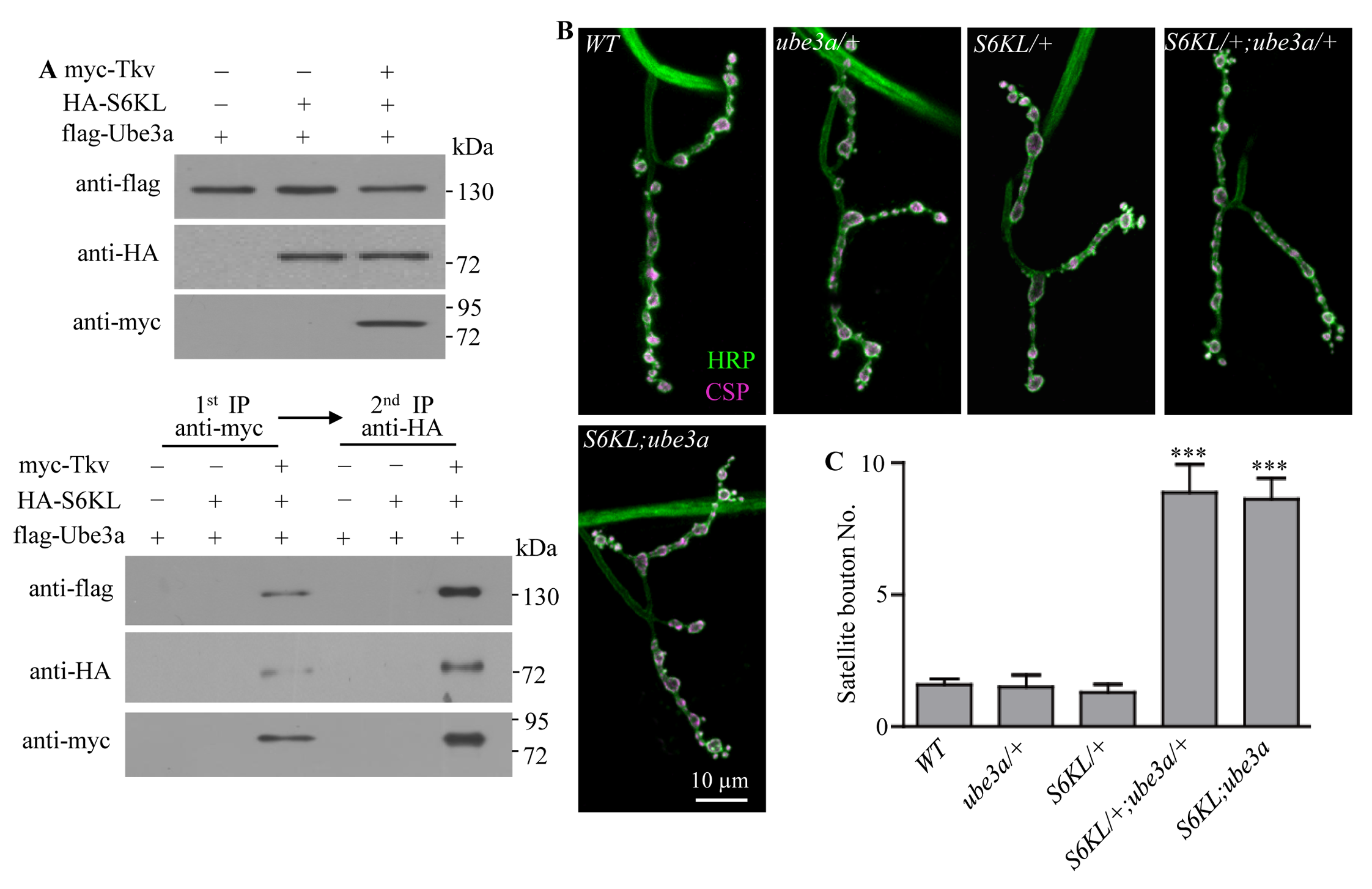

Supplement: S13 Fig — (A) Different plasmid constructs were singly or co-transfected to S2 cells. Transfected cells were harvested 48 hr later. Cell lysates were used in a two-step immunoprecipitation step by anti-myc and anti-HA sequentially. (B) Confocal images of NMJ4 labeled with anti-HRP. Loss of one copy of S6KL or ube3a had no effect on the number of satellite boutons, while the number of satellite boutons increased significantly in S6KL140 and ube3a35 trans-heterozygotes and double mutants. (C) Quantification of satellite bouton number in different genotypes. n ≥ 16 NMJs analyzed, one-way ANOVA, mean ± s.e.m., ***p < 0.001. (TIF) [file pgen.1006062.s013.tif]
