# Supplementary material for: Angelman Syndrome Protein Ube3a Regulates Synaptic Growth and Endocytosis by Inhibiting BMP Signaling in Drosophila
Source: PLoS Genet. 2016 May 27;12(5):e1006062. doi: 10.1371/journal.pgen.1006062 (PMC4883773; doi:10.1371/journal.pgen.1006062)
Supplement: S1 Text — (DOC) [file pgen.1006062.s014.doc]

**Supplementary materials and** **methods**

Electron microscopy (EM) was performed as described previously . Briefly, dissected third-instar larvae were fixed overnight at 4°C in 1% glutaraldehyde plus 4% paraformaldehyde in 0.1M cacodylate buffer, pH 7.4, followed by several rinses with cacodylate buffer. Right and left hemi-segments from abdominal segment A2 or A3 were separated from the larval fillets and post-fixed in 1% OsO4 in cacodylate buffer for 90 min at room temperature. Samples were then stained in saturated aqueous uranyl acetate for 1 h, dehydrated in a graded ethanol series, and embedded in Spurr resin (Electron Microscopy Sciences). Neuromuscular junction 6/7 terminals in abdominal segments A2 and A3 were serially sectioned with a Leica UC6 ultramicrotome, stained with uranyl acetate and Sato’s lead, and observed using a JEOL 1400 electron microscope. For quantification analysis, the number of synaptic vesicles (SVs) within a 200 nm radius of the transmitter release site (T-bar) was determined using ImageJ. Vesicle structures with diameters > 60 nm were defined as cisternae.

**References:**

Shi, W., Chen, Y., Gan, G., Wang, D., Ren, J., Wang, Q., Xu, Z., Xie, W., and Zhang, Y.Q. (2013). Brain tumor regulates neuromuscular synapse growth and endocytosis in Drosophila by suppressing mad expression. J Neurosci *33*, 12352-12363.

Zhao, G., Wu, Y., Du, L., Li, W., Xiong, Y., Yao, A., Wang, Q., and Zhang, Y.Q. (2015). Drosophila S6 Kinase like inhibits neuromuscular junction growth by downregulating the BMP receptor thickveins. PLoS Genet *11*, e1004984.
